# AftD functions as an α1 → 5 arabinofuranosyltransferase involved in the biosynthesis of the mycobacterial cell wall core

**DOI:** 10.1016/j.tcsw.2017.10.001

**Published:** 2017-12-01

**Authors:** Luke J. Alderwick, Helen L. Birch, Karin Krumbach, Michael Bott, Lothar Eggeling, Gurdyal S. Besra

**Affiliations:** a School of Biosciences, University of Birmingham, Edgbaston, Birmingham B15 2TT, UK; b Institute for Biotechnology 1, Forschungszentrum Juelich, D-52425 Juelich, Germany

**Keywords:** *Corynebacterium glutamicum*, *Mycobacterium tuberculosis*, Cell wall, Arabinogalactan, Glycosyltransferase

## Abstract

Arabinogalactan (AG) is an essential structural macromolecule present in the cell wall of *Mycobacterium tuberculosis*, serving to connect peptidoglycan with the outer mycolic acid layer. The D-arabinan segment is a highly branched component of AG and is assembled in a step-wise fashion by a variety of arabinofuranosyltransferases (AraT). We have previously used *Corynebacterium glutamicum* as a model organism to study these complex processes which are otherwise essential in mycobacteria. In order to further our understanding of the molecular basis of AG assembly, we investigated the role of a fourth AraT, now termed AftD by generating single (Δ*aftD*) and double deletion (Δ*aftB* Δ*aftD*) mutants of *C. glutamicum*. We demonstrate that AftD functions as an α(1 → 5) AraT and reveal the point at which it exerts its activity in the AG biosynthetic pathway.

## Highlights

• Generation of a single *aftD* and double *aftB/aftD* mutants in *C. glutamicum*.
• Phenotypic analysis of purified cell wall material indicates a cell wall lesion.
• Cell free biochemical assays indicate AftD functions as an α(1 → 5) AraT.

## Introduction

The *Mycobacterium tuberculosis* cell wall is dominated by a complex heteropolysaccharide termed arabinogalactan (AG) that serves to connect the peptidoglycan (PG) sacculus to the outer ‘myco-membrane’ (Bhowruth et al., 2008). The presence of AG in the cell wall core is a common feature of the *Corynebacteriales*, members of which include Corynebacteria, Nocardia, Rhodobacter and Mycobacteria (Daffe et al., 1993, Dover et al., 2004, Wesener et al., 2017). Although these organisms exist in a variety of different environments, the core structural features common to AG are based upon a common carbohydrate structure.

**Fig. 1A:**
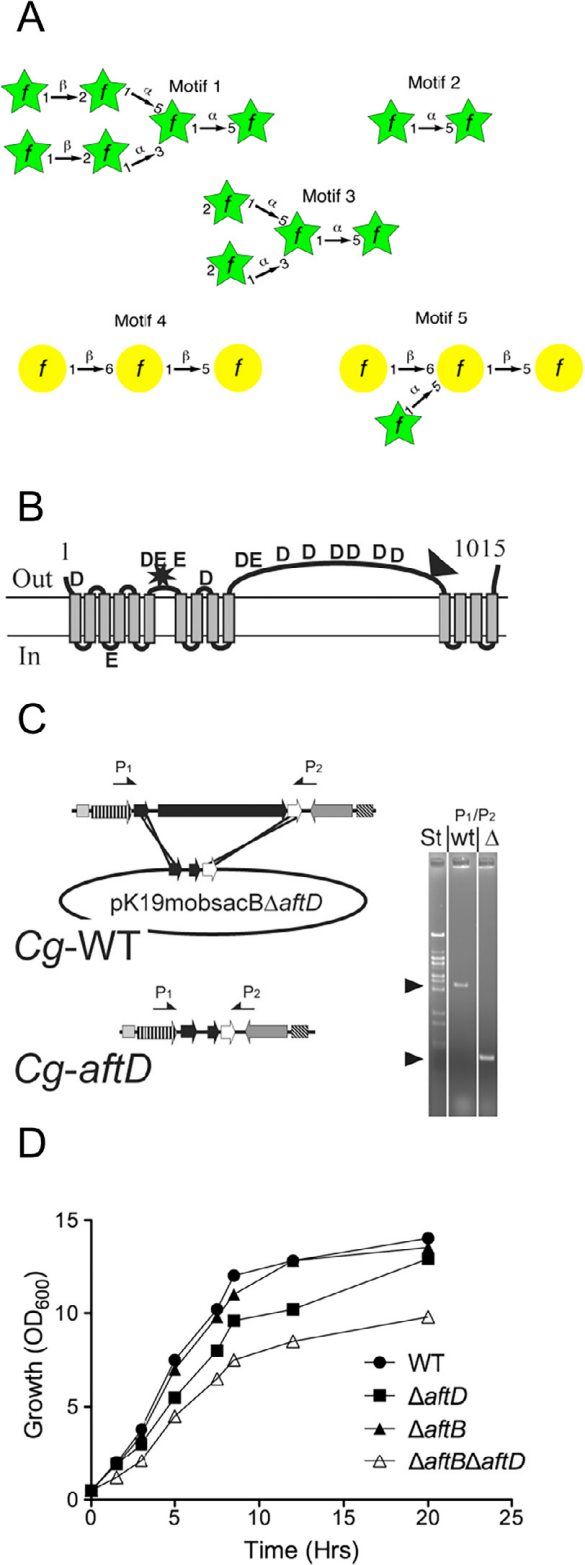
Common glycosyl linkage motifs found within mycobacteria and corynebacteria (A), topology of AftD (B), knock out strategy (C) and effect on growth in liquid media (D). *A*, The hexaarabinosyl (motif) 1 is the site of mycolic acid esterification, the [α-D-Ara*f*(1 → 5]-α-D-Ara*f* (motif 2) represents the main linear segments of D-arabinan, the [α-D-Ara*f*(1 → ]3,5-α-D-Ara*f* (motif 2) illustrates the main bifurcation points of a single D-arabinan chain, and motifs 4 and 5 show the linkage profiles of linear D-galactan configuration of how D-arabinan is attached to D-galactan. *B*, topology of *C. glutamicum* AftD with the black triangle indicating the location of the 388 aa insertion in the *M. tuberculosis* ortholog of AftD. The topology prediction is based on the dense alignment surface method. The conserved aspartyl and glutamyl residues are indicated as D and E, respectively. The star indicates the two catalytic motifs resembling those of glycosyltransferases of the GT-C family (Liu and Mushegian, 2003). *C*, strategy to construct *C. glutamicum*Δ*aftD*. Shown is the wild type genomic *aftD*-region and the deletion vector pK19mobsacBΔ*aftD* carrying 18 nucleotides of the 5′-end of *aftD* and 36 nucleotides of its 3′-end thereby enabling the in-frame deletion of almost the entire Cg-*aftD* gene. Selection for homologous recombination results in *C. glutamicum*Δ*aftD* (*Cg-aftD*). The arrows marked P1 and P2 locate the primers used for the PCR analysis to confirm the absence of Cg-*aftD*. Distances are not drawn to scale. The results of the PCR analysis are shown on the right, where the amplification product obtained from the wild type (WT) and that of the deletion mutant of (Δ) was marked accordingly. The sizes of 4032 bp for the wild type and of 1051 bp for the deletion mutant were as expected and marked by an arrow head. St marks the standard. *D*, the consequences of Cg-*aftD* deletion and Cg-*aftB*/Cg-*aftD* double deletion on growth in rich medium (BHI). Growth of *C. glutamicum* (●), *C. glutamicum*Δ*aftD* (■), *C. glutamicum*Δ*aftD* (▲) and *C. glutamicum*Δ*aftB*Δ*aftD* (Δ).

Interspersed throughout the glycan strands of mycobacterial PG, some of the muramic acid residues serve as sites of AG attachment in which the 6-hydroxyl is covalently bonded *via* a phosphodiester to an N-acetylglucosamine residue, which is α(1 → 3) linked to L-rhamnose, forming a motif commonly known as the linker unit (LU) (McNeil et al., 1990). The LU is attached to a linear galactan polysaccharide composed of approximately 30 galactofuranose (Gal*f*) residues that extend in an alternating β(1 → 5) and β(1 → 6) fashion (Fig. 1A) (Daffé et al., 1990, Daffe et al., 1993, Besra et al., 1995). This galactan “backbone” is decorated with three arabinan chains, which are specifically located at the 8th, 10th and 12th positions (Alderwick et al., 2005). Each arabinan domain is composed of approximately 30 arabinofuranose (Ara*f*) residues that are arranged into a structure that becomes increasingly branched and the overall structure depends entirely upon the arrangement of three major ‘glycosidic motifs’ (Fig. 1A) (Besra et al., 1995). The arabinan polysaccharide is branched by virtue of an α(1 → 3) linked Ara*f* unit positioned at approximately 13 residues along the linear arabinan chain (Besra et al., 1995). The resulting branch point is then extended by an additional stretch of five α(1 → 5) linked Ara*f* residues. Each of these bifurcated strands are again branched in an α(1 → 3) dependent manner before terminating in a characteristic hexasaccharide, [Ara*f*-β(1 → 2)-Ara*f*-α(1 → ]_2_–3,5)-Ara*f*-α(1 → 5)-Ara*f*, often referred to as the ‘hexaarabinofuranosyl motif’ (Besra et al., 1995, Daffé et al., 1990, Daffe et al., 1993, McNeil et al., 1994). A single fully matured arabinan domain provides a total of 8 possible sites for the esterification of cell wall bound mycolates, ultimately forming the mycolyl-ArabinoGalactan-Peptidoglycan (mAGP) complex (McNeil et al., 1991).

Recently, our understanding of the AG biosynthetic pathway has significantly improved. This is largely a result of using surrogate laboratory “model systems” to investigate the deletion of corresponding orthologous genes, which are otherwise essential in mycobacteria. In this regard, *Corynebacterium glutamicum* is an excellent model system for studying mycobacterial cell wall biosynthetic processes (Abrahams and Besra, 2016, Jankute et al., 2015, Alderwick et al., 2006a, Birch et al., 2009). We have characterised several novel arabinofuranosyltransferases (AraTs) which are crucial for the assembly of AG. AftA is responsible for “priming” the galactan core thrice with single Ara*f* residues which is then elongated in an α(1 → 5) processive manner (Alderwick et al., 2006b). The AraT responsible for this activity in *C. glutamicum* is encoded by the single *emb* gene (*NCgl0184*), which if deleted *via* homologous recombination, results in a viable strain that exhibits a severely reduced growth phenotype and a dramatically altered cell wall composition (Alderwick et al., 2005). The *M. tuberculosis* genome encodes for three *Cg-emb* orthologs annotated as *embA*, *embB* and *embC* (with *embC* displaying closest sequence homology of the three) that have been shown to be targeted by the front-line anti-TB drug ethambutol (EMB) (Belanger et al., 1996, Telenti et al., 1997). The terminal β(1 → 2) capping enzyme AftB and the α(1 → 3) branching enzyme AftC were also identified as key enzymes involved in the formation of the non-reducing terminus of AG (Birch et al., 2008, Seidel et al., 2007a). One of the most striking features of these AraTs is that they all belong to the GT-C superfamily of glycosyltransferases. GT-C glycosyltransferases are polytopic integral membrane proteins, often containing an N- or C-terminal “globular” domain which also utilize lipid-linked sugar donors (Liu and Mushegian, 2003). AftD is the fourth member of this GT-C family of AraTs and gene knock out experiments performed in *M. smegmatis* suggested that *aftD* was essential for the viability and growth of *M. smegmatis* (Skovierova et al., 2009). The essential nature of *Ms-aftD* afforded no phenotypic analysis of the mutant, therefore the authors assigned the function of *Ms-aftD* using over-expression experiments, with subsequent biochemical analyses in order to determine what downstream effects this over-expression had on the physiology and chemistry of the cell wall AG (Skovierova et al., 2009). These authors concluded that *aftD* encoded for a second branching α(1 → 3) AraT, similar in function to that of AftC (Birch et al., 2008).

Herein, we report a comprehensive phenotypic characterisation of two modified strains of *C. glutamicum*, one of which has been deleted of its *Mt-aftD* ortholog (*NCgl2757*) in addition to a double mutant which has also been deleted of the terminal β(1 → 2) capping arabinofuranosyltransferase, *aftB*. We present evidence, which provides an alternative explanation for the function of AftD as an α(1 → 5) arabinofuranosyltransferase responsible for elongating the α(1 → 3) primed bifurcation strands of the arabinan found in AG.

## Material and methods

### Strains and culture conditions

*C. glutamicum* strains were grown on complex medium (Brain Heart Infusion, Difco), or on salt medium CGXII (Eggeling and Reyes, 2005), with cultivation at 30 °C. Growth curves were followed from cultures consisting of 50 ml medium in 500-ml baffled Erlenmeyer flasks, which were incubated at 120 rpm and orbital shaking for 50 mm (Kühner, Basel, Switzerland). *Escherichia coli* strains were grown on LB at 37 °C. Kanamycin and ampicillin were used at a concentration of 50 µg/ml and 100 µg/ml respectively. Samples for cell wall analysis were prepared by harvesting cells at an optical density of 10–15 followed by a saline wash and freeze drying (Alderwick et al., 2005).

### Construction of plasmids and strains

The expression vectors made were pVWEx-*Cg-aftD* (*NCgl2757*), pVWEx-*Mt-aftD* (*Rv0236c*) and the deletion vector pK19mobsacBΔ*aftD* (*NCgl2757*) with the gene number of the *C. glutamicum* and *M. tuberculosis aftD* orthologs added in parentheses. To enable in-frame deletion of *aftD* crossover PCR was applied with primer pairs AB (A, 5′-CGCTTCTAGACCACGTCATGGCATACGAAACTG-3′; B, 5′-CCCATCCACTAAACTTAAACACACCACAAAACCCAGCAC-3′) and CD (C, 5′-TGTTTAAGTTTAGTGGATGGGGCTGCGCTATTTGCTGG-3′; D, 5′-GCGGGAATTCGCATGGCAAGCCGGTAAG-3′) and genomic DNA of the wild type of *C. glutamicum* (ATCC13032) used as template. Both amplified products were used in a second PCR with primer pairs AD to generate a 620 bp fragment consisting of sequences adjacent to *Cg*-*aftD*, which was ligated with XbaI-EcoRI-cleaved pK19mobsacB.

For the construction of pVWEx-*Mt-aftD M. tuberculosis* H37Rv DNA was used as template obtained from the Tuberculosis Research Material Contract (National Institutes of Health) at Colorado State University. Since the direct amplification of the 4203 bp *aftD* gene product proofed difficult, crossover PCR was applied with primer pairs EF (E, 5′-TACCGAGCTCGAATTCAAGGAGATATAGATGTGGCGCCGTTGTCTCGCAAATG-3′; F, 5′-CGAGGAGCTCGACACGGTGATC-3′) and GH (G, 5′-GACGGCCAGTGAATTCCTATTGCATGCGGTCTTGACCACGAGACTC-3′; H, 5′-GATCACCGTGTCGAGCTCCTCG-3′), using the pair EG in the second PCR. The resulting product was cloned into pEKEx2 using poxvirus DNA polymerase of the In-Fusion kit (Clontech, Takara Bio Inc, Japan). To construct pVWEx-*Cg*-*aftD*, the primer pairs 5′-GATTAACTGCAGAGGGAGATATAGGTGCTGGGTTTTGTGG-3′ and 5′-GCCGTCTCTAGATTAGCGCTTTGGAGGCC-3′ were used to amplify *NCgl2757* plus 4 additional codons at the 5‘-end. The product was purified and ligated with PstI-XbaI-treated pVWEx1.

All plasmid inserts were confirmed by sequencing. The chromosomal deletion of *Cg-aftD* was performed as described previously using two rounds of positive selection (Schafer et al., 1994), and its successful deletion was verified by use of two different primer pairs hybridizing in the genome. Plasmids were introduced into *C. glutamicum* by electroporation with selection to kanamycin resistance (25 µg/ml) on BHI. In order to construct the *aftB*/*aftD* double mutant *C. glutamicum*Δ*aftB*Δ*aftD*, the deletion vector pK19mobsacBΔ*aftD* was also used and introduced into the previously reported strain *C. glutamicum*Δ*aftB* (Seidel et al., 2007a).

### Isolation of the mAGP complex, glycosyl composition and linkage analysis of alditol acetates by GC and GC/MS

The thawed cells were resuspended in phosphate buffered saline containing 2% Triton X-100 (pH 7.2), disrupted by sonication and centrifuged at 27,000×*g* (Alderwick et al., 2005, Besra et al., 1995). The pelleted material was extracted three times with 2% SDS in phosphate buffered saline at 95 °C for 1 h, washed with water, 80% (v/v) acetone in water, and acetone, and finally lyophilised to yield a highly purified cell wall preparation (Alderwick et al., 2005, Besra et al., 1995). Cell wall or per-*O*-methylated cell wall preparations (Alderwick et al., 2005) were hydrolyzed in 2 M TFA, reduced with NaB^2^H_4_ and the resultant alditols per-*O*-acetylated and examined by GC and GC/MS as described previously (Alderwick et al., 2005, Besra et al., 1995).

### Arabinofuranosyltransferase activity with membrane preparations of *C. glutamicum*, *C. glutamicum*Δ*aftB*, *C. glutamicum*Δ*aftD* and *C. glutamicum*Δ*aftB*Δ*aftD*

Membranes were prepared as described previously (Birch et al., 2008, Lee et al., 1997, Seidel et al., 2007a) and resuspended in 50 mM MOPS (pH 7.9), containing 5 mM β-mercaptoethanol and 10 mM MgCl_2_ (buffer A) to a final protein concentration of 15–10 mg/ml. The neoglycolipid acceptors used in this study were α-D-Ara*f*(1 → 3)-α-D-Ara*f*-*O*-(CH_2_)_7_CH_3_ (acceptor A) and a branched trisaccharide [α-D-Ara*f*(1 → ]3,5-α-D-Ara*f*-*O*-(CH_2_)_7_CH_3_ (acceptor B) (Lee et al., 1997). The acceptors (A and B) (Lee et al., 1997) (stored in ethanol) and decaprenol monophosphate (stored in CHCl_3_/CH_3_OH, 2:1. v/v) were aliquoted into 1.5 ml eppendorf tubes to a final concentration of 2 mM and 5 μg/ml, respectively, and dried under compressed nitrogen. The arabinofuranosyltransferase assay was carried out as described previously (Lee et al., 1997) with modifications. IgePal™ (Sigma–Aldrich) was added (0.1%, v/v) with the appropriate amount of buffer A (final volume 80 μl). Tubes were sonicated for 15 min to resuspend lipid linked substrates and then mixed with the remaining assay components, which included membrane protein from either *C. glutamicum*, *C. glutamicum*Δ*aftB*, *C. glutamicum*Δ*aftD* and *C. glutamicum*Δ*aftB*Δ*aftD* (1 mg), 1 mM ATP, 1 mM NADP and in some cases EMB (0.1 mg/ml). Assays were initiated with the addition of 100,000 cpm p[^14^C]Rpp and incubated for 2 h at 37 °C and quenched by the addition of 533 μl CHCl_3_/CH_3_OH (1:1, v/v). After mixing and centrifugation at 27,000×*g* for 15 min at 4 °C, the supernatant was removed and dried under nitrogen. The residue was then resuspended in 700 μl of CH_3_CH_2_OH/H_2_O (1:1, v/v) and loaded onto a 1 ml SepPak strong anion exchange cartridge (Supelco), pre-equilibrated with CH_3_CH_2_OH/H_2_O (1:1, v/v). The column was washed with 2 ml CH_3_CH_2_OH and the eluate collected, dried and partitioned between the two phases arising from a mixture of *n*-butanol (3 ml) and water (3 ml). The resulting organic phase was recovered following centrifugation at 3500×*g* and the aqueous phase again extracted twice with 3 ml of water-saturated *n*-butanol. The pooled extracts were back-washed twice with *n*-butanol-saturated water (3 ml). The *n*-butanol fraction was dried and resuspended in 200 μl butanol. The extracted radiolabelled material was quantified by liquid scintillation counting using 10% of the labelled material and 5 ml of EcoScintA (National Diagnostics, Atlanta). The incorporation of [^14^C]Ara*f* was determined by subtracting counts present in control assays (incubations in the absence of acceptor). The remaining labelled material was subjected to thin-layer chromatography (TLC) using isopropanol:acetic acid:water (8:1:1, v/v/v) on aluminium-backed Silica Gel 60 F_254_ plates (Merk, Darmstadt, Germany). Autoradiograms were obtained by exposing TLCs to X-ray film (Kodak X-Omat) for 3 days.

### Characterisation of AftD responsible for α(1 → 5)-arabinofuranosyltransferase activity with membranes prepared from *C. glutamicum*, *C. glutamicum*Δ*aftB*, *C. glutamicum*Δ*aftD* and *C. glutamicum*Δ*aftB*Δ*aftD*

Large-scale reaction mixtures containing cold DPA (200 μg, 0.75 mM) (Lee et al., 1997) and 50 mM of either acceptor A or acceptor B, were mixed and given an initial 1 h incubation at 37 °C with membranes prepared from either *C. glutamicum*, *C. glutamicum*Δ*aftB*, *C. glutamicum*Δ*aftD*, *C. glutamicum*Δ*aftB*Δ*aftD* and *C. glutamicum*Δ*aftB*Δ*aftD* pVWEx-*Mt-aftD*. The assays were replenished with fresh membranes (1 mg) and re-incubated for 1 h at 37 °C with the entire process repeated thrice. Products were extracted from reaction mixtures by *n*-butanol/water phase separation as described earlier to extract products. Products were applied to preparative TLC plates, developed in isopropanol:acetic acid:water (8:1:1, v/v/v) and sprayed with 0.01% 1,6-diphenylhexatriene in petroleum-ether:acetone (9:1, v/v), and the products localized under long-wave (366 nm) UV light (Lee et al., 1997). The plate was then re-developed in toluene to remove the reagent and the bands recovered from the plates by extraction with *n*-butanol. The butanol phases were washed with water saturated with *n*-butanol and the dried products subjected to GC/MS as described (Lee et al., 1997, Alderwick et al., 2006b) and analysed by electrospray mass spectrometry (ES-MS) in the positive mode on a Micromass LCT mass spectrometer as described previously (Lee et al., 1997).

## Results

### Genome comparison of the *aftD* locus

An *in silico* analysis of the *Rv0236c* open reading frame from *M. tuberculosis* as well as its ortholog (*NCgl2757*) from *C. glutamicum* revealed a conserved syntenic region which is present in all species belonging to the order *Corynebacteriales* (Fig. S1). Our *in silico* analyses support the initial report of Skovierova et al. (2009) who originally annotated this gene by the acronym *aftD* (arabinofuranosyltransferase D).

The *C. glutamicum* AftD (*Cg*-AftD) variant is a polytopic membrane protein of 1015 aa. TMHMM predictive modelling indicates that *Cg*-AftD possesses 14 transmembrane α-helices (TMHs) (Sonnhammer et al., 1998) with a long extended loop of 510 aa separating the first 10 TMHs of the protein from the final 4 C-terminal TMHs (Fig. 1B). The various residues contributing to AftD catalytic activity are expected to reside within the N-terminal region of the protein. Indeed, a conserved glycosyltransferase motif, DX_3_DX_18_D, characteristic for the GT-C family is located immediately after TMH6 and is marked by a star (Fig. 1B) (Liu and Mushegian, 2003). Interestingly, a second structurally related motif, DX_2_EX_12_E, is present between THM10 and TMH11. The C-terminal half of *Cg*-AftD is predicted to contain a carbohydrate-binding module (CBM) at position 619–740. Several examples of proteins containing such CBM domains, include the periplasmic polygalacturonic acid binding protein from *Yersinia enterocolitica* (Abbott et al., 2007), a lectin-related virulence factor of *Streptococcus pneumoniae* (Boraston et al., 2006) and perhaps more significantly, the C-terminal CBM domain of the *M. tuberculosis* EmbC^CT^ (Alderwick et al., 2011). The *Mt*-AftD protein is larger than its corynebacterial counterpart (*Cg*-AftD) by up to 388 aa, which is due to an insert in the loop region (707–830 in *Mt*-AftD), as marked by a black triangle (Fig. 1B). An additional insertion in *Mt*-AftD (position 1022-1253) represents another CBM similar to that of the cyclodextrin glycosyltransferase present in *Bacillus circulans* (Uitdehaag et al., 1999).

### Construction and growth of mutants

The non-replicative vector pK19mobsacBΔ*aftD* was used to transform *C. glutamicum* to kanamycin resistance, indicating integration in its chromosome. For selection of the second recombination event clones were cultivated on sucrose, and 12 Suc^R^-Kan^S^ clones analysed *via* PCR with the primer pair P1/P2 (Fig. 1C). This resulted in 4 clones with a 4032 bp fragment as expected for the wild type, whereas 8 clones afforded a 1051 bp fragment and deletion of *aftD*. One deletion mutant was termed *C. glutamicum*Δ*aftD*. In addition, *aftD* was also deleted in strain *C. glutamicum* Δ*aftB* (Seidel et al., 2007a) to achieve the double mutant *C. glutamicum*Δ*aftB*Δ*aftD*. Growth of *C. glutamicum*Δ*aftD* on the salt medium CGXII was not influenced (data not shown), whereas on the complex medium BHI (Difco) a significantly reduced growth rate of 0.48 h^−1^ was observed as compared to wild type exhibiting a growth rate of 0.62 h^−1^ (Fig. 1D). Upon reintroduction of *aftD* in the deletion mutant, as was the case in *C. glutamicum*Δ*aftD* pVWEx-*Cg*-*aftD*, growth was fully restored (Fig. S2A). Interestingly, with pVWEx-Mt-*aftD* a negative effect on growth was observed which we interpret as expression of the mycobacterial ortholog, potentially causing some incomplete integration of the protein into the membrane of the heterologous host (Fig. S2A). The double mutant *C. glutamicum*Δ*aftB*Δ*aftD* exhibited an even further reduced growth rate of 0.40 h^−1^, whereas the growth rate of the single mutant *C. glutamicum*Δ*aftB* was unaffected (Fig. 1D). The complemented double mutants, expressing either Cg-*aftD* or Mt-*aftD*, revealed the former with a growth phenotype similar to *C. glutamicum*Δ*aftB* and the latter having little positive restorative effect (Fig. S2B).

### Cell wall lipid analysis of *C. glutamicum* and mutant strains

After generating a variety of *C. glutamicum* mutant and complemented strains, we conducted a phenotypic analysis of the lipid profiles exhibited by *C. glutamicum*, *C. glutamicum*Δ*aftB*, *C. glutamicum*Δ*aftD*, *C. glutamicum*Δ*aftD* pVWEx-*Cg*-*aftD*, *C. glutamicum*Δ*aftD* pVWEx-*Mt*-*aftD*, *C. glutamicum*Δ*aftB*Δ*aftD*, *C. glutamicum*Δ*aftB*Δ*aftD* pVWEx-*Cg*-*aftD* and *C. glutamicum*Δ*aftB*Δ*aftD* pVWEx-*Mt*-*aftD.* Each of these eight strains was cultured to mid-log phase in complex liquid media before pulse labelling with [^14^C]-acetic acid and the “free” and “cell wall bound” corynemycolic acid profiles analysed as described previously (Alderwick et al., 2006b, Birch et al., 2008, Seidel et al., 2007a, Seidel et al., 2007b). Thin layer chromatography (TLC) analysis revealed some subtle, yet important, alterations in the profile of the extractable “free lipids” (Fig. S3A). The lipid profile obtained for *C. glutamicum*Δ*aftB* is consistent with previously published results, whereby an accumulation of trehalose monocoronomycolate (TMCM) occurs (Seidel et al., 2007a). Similarly, an increase in TMCM can also be observed for *C. glutamicum*Δ*aftD* and *C. glutamicum*Δ*aftB*Δ*aftD* (Fig. S3A). This is an interesting observation since we have previously observed an almost identical alteration in the phenotype of the extractable free lipids for similar AraT knock outs in *C. glutamicum*, such as *emb* (Alderwick et al., 2005), *aftA* (Alderwick et al., 2006b), *aftB* (Seidel et al., 2007a) and also in *M. smegmatis* deleted of *aftC* (Birch et al., 2008). Table 1 summarises the alterations in the composition cell wall lipids of the mutant strains relative to the *C. glutamicum* wild type strain.

**Table 1:** Comparative analysis and alteration in cell wall lipid, monosaccharide and glycosyl linkage composition from *C. glutamicum*, *C. glutamicum*Δ*aftB*, *C. glutamicum*Δ*aftD*, *C. glutamicum*Δ*aftB*Δ*aftD*, *C. glutamicum*Δ*aftD* pVWEx-*Cg*-*aftD*, *C. glutamicum*Δ*aftD* pVWEx-*Mt*-*aftD*, *C. glutamicum*Δ*aftB*Δ*aftD* pVWEx-*Cg*-*aftD* and *C. glutamicum*Δ*aftB*Δ*aftD* pVWEx-*Mt*-*aftD*.

	*C. glutamicum*	*C. glutamicum*Δ*aftB*	*C. glutamicum*Δ*aftD*	*C. glutamicum*Δ*aftB*Δ*aftD*	*C. glutamicum*Δ*aftD* + pVWEX-*Cg-aftD*	*C. glutamicum*Δ*aftD* + pVWEX-*Mt-aftD*	*C. glutamicum*Δ*aftB*Δ*aftD* + pVWEX-*Cg-aftD*	*C. glutamicum*Δ*aftB*Δ*aftD* + pVWEX-*Mt-aftD*
Change in TDCM (%)	–	−52.3	−2.3	+74.5	−51.9	−1.9	−30.4	−59.4
Change in TMCM (%)	–	+29.6	+50.4	+15.0	+30.2	+50.9	+42.6	+58.1
Change in CMAME (%)	–	−34.4	−12.0	−60.5	−25.4	−25.0	−48.1	−58.4
Ratio of Ara:Gal	2.75:1	2.8:1	2.55:1	3.06:1	2.76:1	2.46:1	2.85:1	2.74:1
Change in Ara (%)	–	+1.8	−7.3	+11.3	−3.3	−5.1	+75	+41
t-Ara*f*	11.4	18.5	10.4	9.9	10.9	10.5	19.1	19.4
t-Rha*p*	11.3	12.6	12.0	10.6	12.1	11.3	11.8	11.8
2-Ara*f*	10.6	0	10.5	–	10.5	10.9	0	0
3-Ara*f*	–	–	–	5.7	–	–	–	–
5-Ara*f*	22.7	23.7	20.8	32	23.5	21.8	23.2	22.2
t-Gal*f*	1.5	1.5	1.6	1.4	1.5	1.6	1.5	1.4
3,5-Ara*f*	9.1	9.6	9.5	9.2	9.1	8.3	8.9	10.4
2,5-Ara*f*	11.4	12.6	12.0	10.6	10.5	11.3	14.0	12.5
5-Gal*f*	9.1	8.9	9.6	8.5	9.1	9.8	8.9	8.3
6-Gal*f*	7.6	7.4	8.0	7.1	7.5	8.3	7.3	6.9
5,6-Gal*f*	5.3	5.2	5.6	5.0	5.3	6.0	5.1	6.9

Cell wall bound corynemycolates were released from the delipidated cells of each strain under investigation and subsequently chemically modified to produce corynemycolic acid methyl esters (CMAME) and subjected to TLC and densitometry analysis (Fig. S3B). As summarised in Table 1, there was a marked reduction in the band corresponding to corynemycolates esterified to cell wall AG in the *aftB* deletion strain (reduced by ∼70%) which was barely affected in the Δ*aftD* strain. In comparison to *C. glutamicum*Δ*aftB*, *C. glutamicum*Δ*aftB*Δ*aftD* displayed a further reduction in CMAMEs which when complemented with a plasmid expressing *aftD* from either *C. glutamicum* (*Cg*-*aftD*) or *M. tuberculosis* (*Mt-aftD*), failed to restore the phenotype to the expected levels similar to that of a Δ*aftB* strain (Fig. S3B). In order to make a proper interpretation of these cell wall lipid phenotypes, we continued our study of these mutant strains by investigating the sugar composition of the cell wall AG.

### Compositional analysis of arabinogalactan isolated from *C. glutamicum* and *C. glutamicum* mutant strains

Highly purified mAGP cell wall material was isolated from the following strains, *C. glutamicum*, *C. glutamicum*Δ*aftB*, *C. glutamicum*ΔaftD, *C. glutamicum*ΔaftBΔaftD, *C. glutamicum*Δ*aftD* pVWEx-*Cg*-*aftD*, *C. glutamicum*Δ*aftD* pVWEx-*Mt*-*aftD*, *C. glutamicum*Δ*aftB*Δ*aftD* pVWEx-*Cg*-*aftD* and *C. glutamicum*Δ*aftB*Δ*aftD* pVWEx-*Mt*-*aftD*. The purified mAGP was then chemically derivatized to alditol acetates and analysed by gas chromatography (GC) (Fig. S4) (Alderwick et al., 2005). The mAGP carbohydrate composition of all eight *C. glutamicum* strains (from Fig. S4) are summarised in Table 1. No significant change in the Ara:Gal ratio could be detected for *C. glutamicum*Δ*aftB* (Seidel et al., 2007a). However, in the case of *C. glutamicum*Δ*aftD*, a 7.3% decrease in the content of Ara in the isolated AG was observed. Due to only a slight reduction in the Ara content in *C. glutamicum*Δ*aftD*, it is difficult to make any clear statement regarding the phenotype of *C. glutamicum*Δ*aftD* upon complementation with a plasmid expressing either *Cg-aftD* or *Mt-aftD*. However, our data suggests that complementation with either *Cg-aftD* or *Mt-aftD* restored the mutant phenotype to almost that of a wild type situation (Ara:Gal ratio of 2.76:1 and 2.46:1, respectively). Interestingly, the glycosyl compositional analysis of the double mutant *C. glutamicum*Δ*aftB*Δ*aftD*, afforded a significant relative increase in the content of cell wall Ara (+11.3%). Furthermore, only a moderate alteration of this phenotype occurred upon the reintroduction of *Cg-aftD* or *Mt-aftD* into *C. glutamicum*Δ*aftB*Δ*aftD* which mirrors the observations regarding the CMAME analysis of these complemented strains (Fig. S3B, Table 1). In order to establish the precise function of AftD, in terms of its role as a cell wall biosynthetic GT-C glycosyltransferase, we investigated the glycosidic linkage profiles of AG isolated from each of the *C. glutamicum* strains.

### Structural characterisation of AG isolated from *C. glutamicum* and mutant strains

**Fig. 2:**
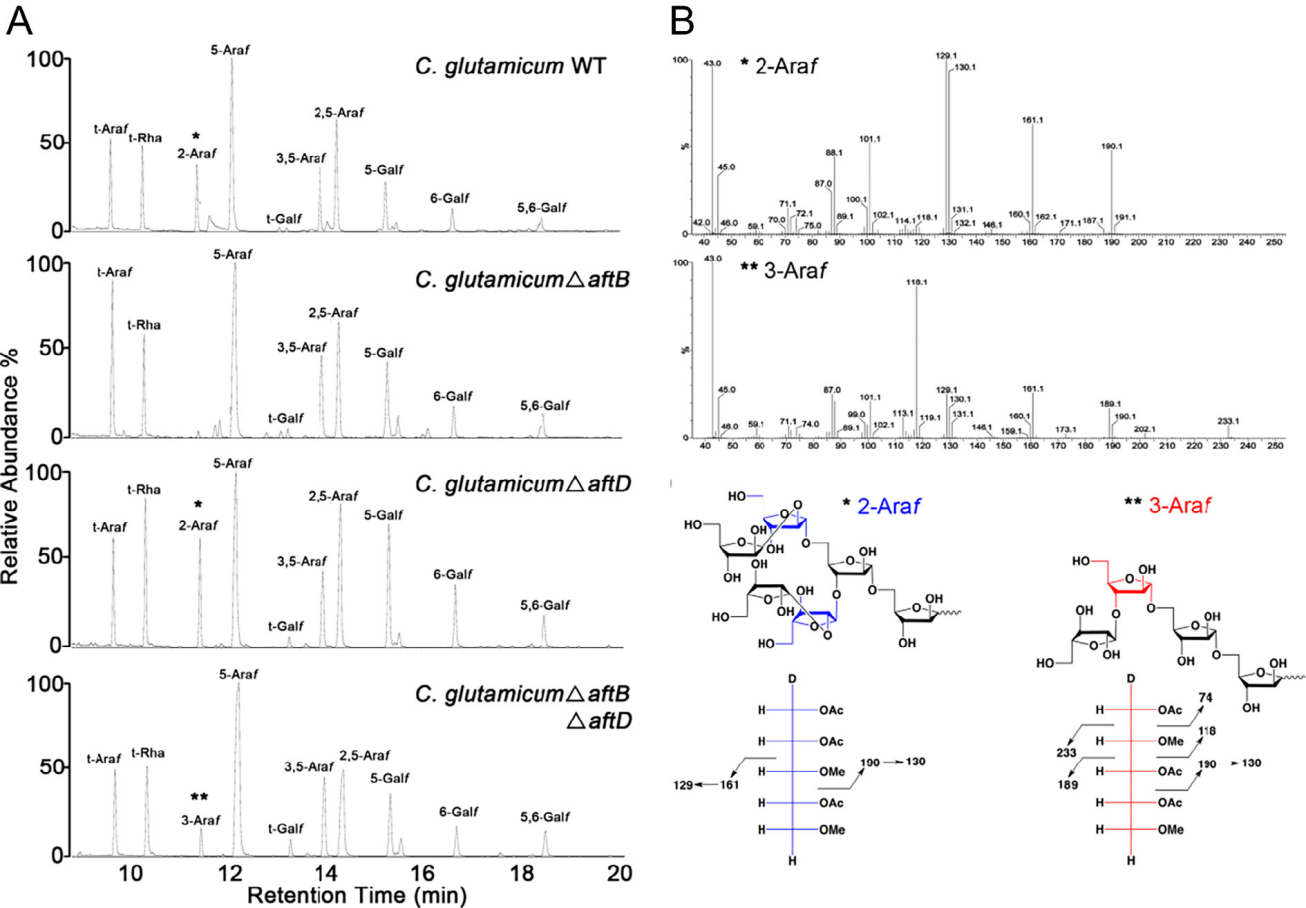
Gas Chromatography/Mass Spectrometry (GC/MS) analysis of partially per-*O*-methylated, per-*O*-acetylated alditol acetate derivatives of purified arabinogalactan from *C. glutamicum*, *C. glutamicum*Δ*aftB*, *C. glutamicum*Δ*aftD* and *C. glutamicum*Δ*aftB*Δ*aftD*. *A*, gas chromatograms demonstrating the presence of glycosyl linkages in purified AG. *B*, mass spectra fragmentation profile of peaks resolving at 11.3 min corresponding to 2-Ara*f* (^*^) and 3-Ara*f* (^**^) and cleavage ions representing fragmentation of 2-Ara*f* (^*^) and 3-Ara*f* (^*^) located in terminal arabinan glycosyl motifs.

Gas chromatography mass spectrometry (GC/MS) was used to analyze the partially *O*-methylated alditol acetate derivatives of mAGP isolated from *C. glutamicum*, *C. glutamicum*Δ*aftB*, *C. glutamicum*Δ*aftD* and *C. glutamicum*Δ*aftB*Δ*aftD* (Fig. 2A, Table 1). The AG isolated from *C. glutamicum*Δ*aftB* was completely devoid of any terminal β(1 → 2) linked Ara*f* residues (Seidel et al., 2007a). Glycosyl linkage analysis from AG isolated from *C. glutamicum*Δ*aftD* suggested only a moderate reduction in the peak at a RT of 12.1 min, which corresponds to a slight decrease in 5-Ara*f* glycosidic linkages (Fig. 2A, Table 1). The abundance of all other peaks, which represent the remaining glycosidic linkages remain unchanged (when compared to the WT profile) (Fig. 2A, Table 1). Here, it is important to note that the 2-Ara*f* peak is present in *C. glutamicum*Δ*aftD*, as confirmed by the mass fragmentation profile illustrated in Fig. 2B. Reintroduction of a plasmid encoding either Cg-*aftD* or Mt-*aftD* into *C. glutamicum*Δ*aftD* resulted in a glycosidic linkage phenotype almost identical to that of the wild type (Fig. S5 and Table 1). Interestingly, the GC/MS profile of the *C. glutamicum*Δ*aftB*Δ*aftD* mutant displays some unique properties as compared to the other three profiles as illustrated in Fig. 2A. In addition to the loss of 2-Ara*f* (due to the deletion of *Cg*-*aftB*), an 11% increase in 5-Ara*f* residues can be observed in the *C. glutamicum*Δ*aftB*Δ*aftD* double mutant as compared to the wild type (Fig. 2A and Table 1). Most interesting is the apparent re-appearance of a peak with a retention time similar to 2-Ara*f* (11.5 min), but with a completely different mass fragmentation pattern (Fig. 2B). This particular residue is unique to *C. glutamicum*Δ*aftB*Δ*aftD*, and the data support a 3-Ara*f* unit (Fig. 2B). Transformation of *C. glutamicum*Δ*aftB*Δ*aftD* with plasmids expressing either *Cg*-*aftD* or *Mt*-*aftD*, restored the *aftB/aftD* double deletion phenotype to one which is identical to that of *C. glutamicum*Δ*aftB*, thus demonstrating that *Cg*-*aftD* and *Mt*-*aftD* both have an equal capacity for complementation of the chromosomally deleted version of *Cg*-*aftD* (Fig. S5 and Table 1). These results strongly support the notion that both *Cg*-*aftD* and *Mt*-*aftD* are directly involved in some aspect of D-arabinan cell wall biosynthesis, most likely through an AraT enzyme with a distinctive α(1 → 5) transferase activity. To further corroborate our hypothesis that AftD is an α(1 → 5) AraT, we performed individual ^1^H, ^13^C HSQC 2D-NMR scans of highly purified base solubilised AG isolated from *C. glutamicum* WT, *C. glutamicum*Δ*aftB*, *C. glutamicum*Δ*aftD* and *C. glutamicum*Δ*aftB*Δ*aftD* (Fig. S6). Interpretation of the expanded anomeric region obtained from *C. glutamicum* revealed a complex pattern of spin systems linked to the AG polysaccharide (Fig. S6A). By relating these data to similarly published information (Birch et al., 2008, Lee et al., 2005, Liu et al., 2011, Mahrous et al., 2008), we fully assigned each of the resonances I-X (Fig. S6A). As expected, resonances corresponding to positions I, II and III are absent in the spectra obtained from AG isolated from *C. glutamicum*Δ*aftB*, confirming a complete loss of terminal β(1 → 2)-Ara*f* residues at the non-reducing end of the arabinan domain (Fig. S6B). In terms of AG prepared from *C. glutamicum*Δ*aftD*, initial inspection of the spectra (in comparison to the WT spin systems) indicates a full complement of residues present within the AG of this mutant, which is similar to the earlier GC/MS results (Fig. 2A). However, a slight reduction in the volume and intensity of peak IV (5-Ara*f*, spin system δ 111.01 ppm/δ 5.03 ppm) is evident. Taken together the GC/MS data (Fig. 2), lends further support to our hypothesis that AftD is an α(1 → 5) arabinofuranosyltransferase. Analysis of AG spectra from *C. glutamicum*Δ*aftB*Δ*aftD* highlights two noteworthy differences when compared to the WT spin systems. First, there is a prominent increase in the volume and intensity of peak IV (5-Ara*f*, spin system δ 111.01 ppm/δ 5.03 ppm) relating to a large increase in the overall abundance of Ara*f* in the AG isolated from *C. glutamicum*Δ*aftB*Δ*aftD* (Fig. S6D). This result also correlates directly with our data describing a large increase in Ara*f* from the total sugar compositional analysis of *C. glutamicum*Δ*aftB*Δ*aftD* (Fig. 2 and Table 1). Second, the new spin system XI (δ 110.6 ppm/δ 5.11 ppm) is also clearly present, which we have assigned as corresponding to the introduction of an α-3-Ara*f* linkage (Fig. S6D). Collectively, our chemical analysis of AG isolated from both *C. glutamicum*Δ*aftD* and *C. glutamicum*Δ*aftB*Δ*aftD* mutants implicates AftD as an α(1 → 5) AraT.

### *In vitro* arabinofuranosyltransferase activity from cell-free extracts of *C. glutamicum* and mutant strains using neo-glycolipid acceptors and p[^14^C]Rpp

**Fig. 3:**
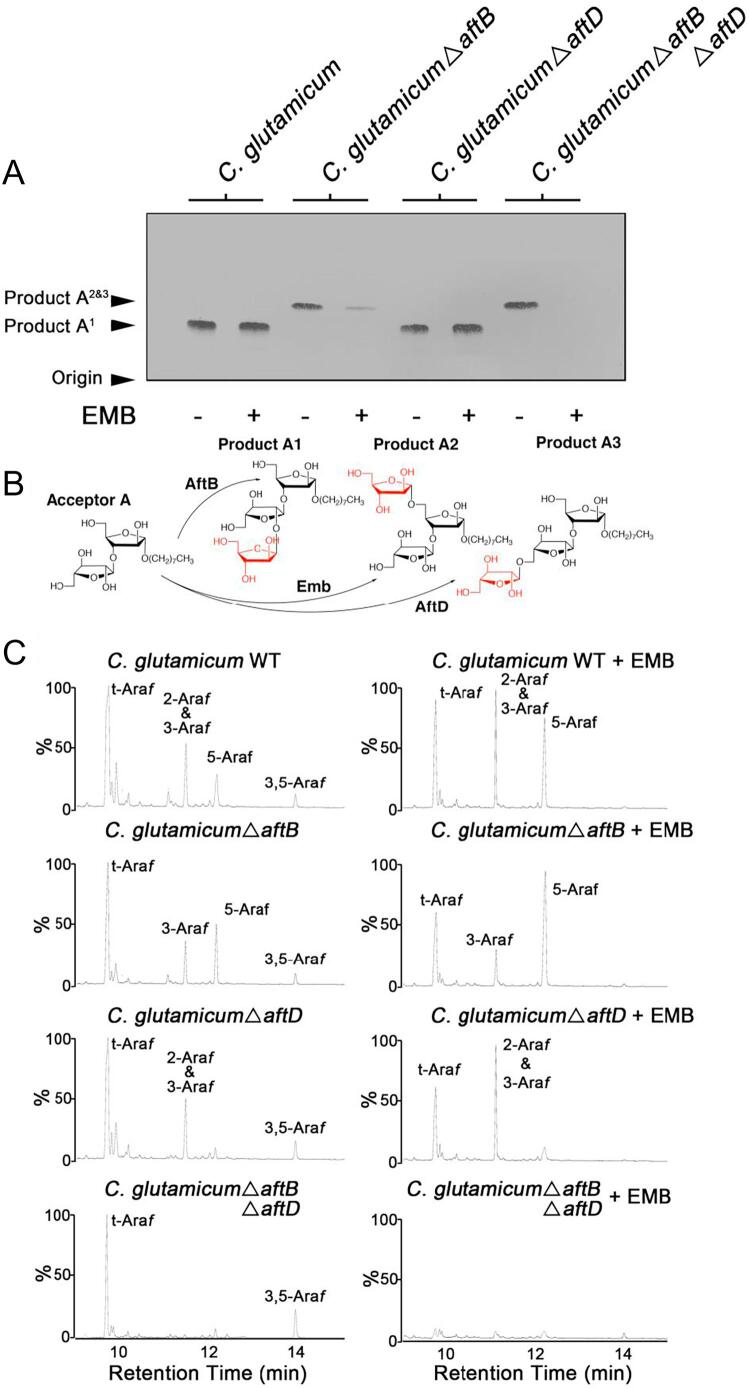
Arabinofuranosyltransferase activity utilizing neoglycolipid acceptor A and membranes prepared from *C. glutamicum*, *C. glutamicum*Δ*aftB*, *C. glutamicum*Δ*aftD* and *C. glutamicum*Δ*aftB*Δ*aftD. A*, Arabinofuranosyltransferase activity was determined using the synthetic neoglycolipid acceptor α-D-Ara*f*(1 → 3)-α-D-Ara*f*-*O*-(CH_2_)_7_CH_3_ (acceptor A) in a cell-free assay with and without EMB (100 μg/ml) as previously described (Lee et al., 1997). The products of the assay were resuspended prior to scintillation counting (10%) and the remaining subjected to TLC using silica gel plates (5735 silica gel 60F_254_, Merck) in isopropanol:acetic acid:water (8:1:1, v/v/v) with the reaction products visualized by autoradiography. The TLC autoradiogram is representative of several independent experiments. *B*, biosynthetic reaction scheme of products A^1^, A^2^ and A^3^ formed in arabinofuranosyltransferase assays using the neoglycolipid acceptor A. *C*, GC/MS analysis of the partially per-*O*-methylated, per-*O*-acetylated alditol acetate derivative of reaction products obtained from assays containing membranes prepared from either *C. glutamicum*, *C. glutamicum*Δ*aftB*, *C. glutamicum*Δ*aftD* and *C. glutamicum*Δ*aftB*Δ*aftD.*

**Fig. 4:**
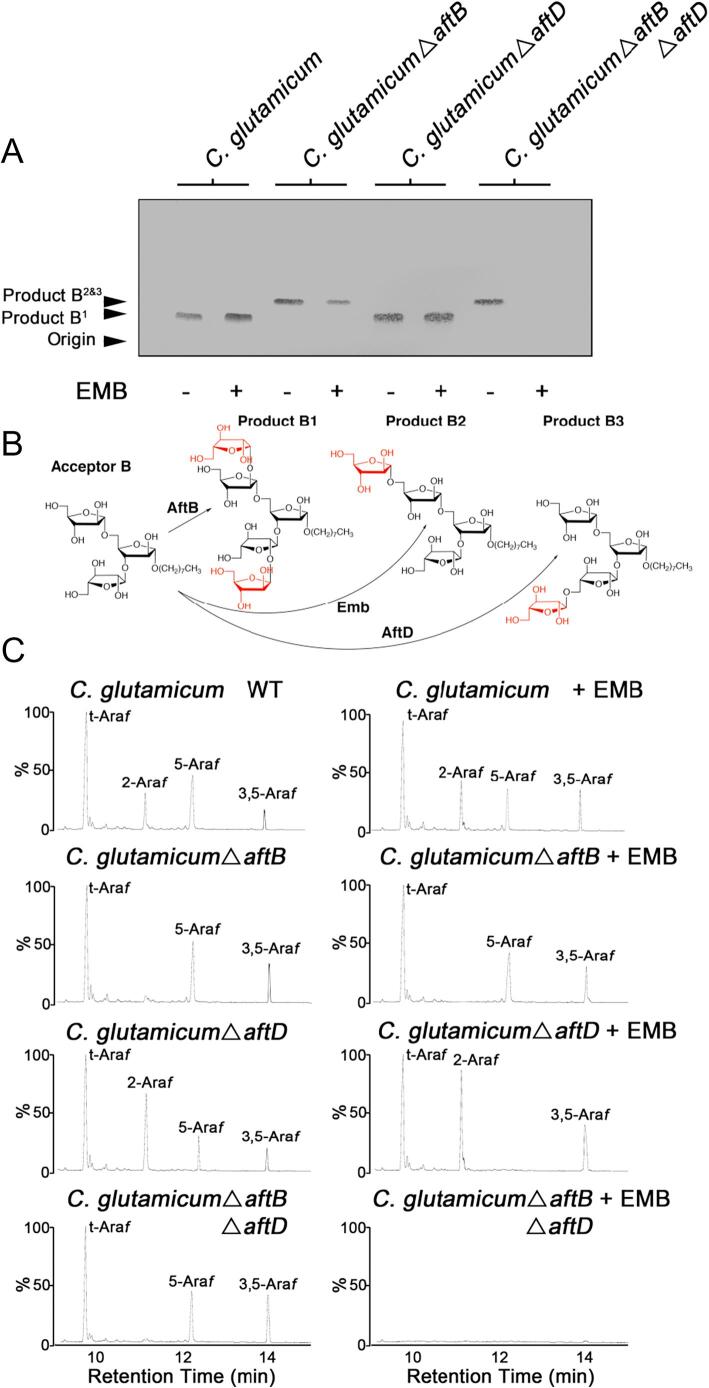
Arabinofuranosyltransferase activity utilizing neoglycolipid acceptor B and membranes prepared from *C. glutamicum*, *C. glutamicum*Δ*aftB*, *C. glutamicum*Δ*aftD* and *C. glutamicum*Δ*aftB*Δ*aftD*. *A*, Arabinofuranosyltransferase activity was determined using the synthetic neoglycolipid acceptor [α-D-Ara*f*(1 → ]3,5-α-D-Ara*f*-*O*-(CH_2_)_7_CH_3_ (acceptor B) in a cell-free assay with and without EMB (100 μg/ml) as previously described (Lee et al., 1997). The products of the assay were resuspended prior to scintillation counting (10%) and the remaining subjected to TLC using silica gel plates (5735 silica gel 60F_254_, Merck) in isopropanol:acetic acid:water (8:1:1, v/v/v) with the reaction products visualized by autoradiography. The TLC autoradiogram is representative of several independent experiments. *B*, Biosynthetic reaction scheme of products B^1^, B^2^ and B^3^ formed in arabinofuranosyltransferase assays using the neoglycolipid acceptor B. *C*, GC/MS analysis of the partially per-*O*-methylated, per-*O*-acetylated alditol acetate derivative of reaction products obtained from assays containing membranes prepared from either *C. glutamicum*, *C. glutamicum*Δ*aftB*, *C. glutamicum*Δ*aftD* and *C. glutamicum*Δ*aftB*Δ*aftD.*

We selected two neo-glycolipid acceptors to use as molecular probes for analysing AraT activity, a linear disaccharide α-D-Ara*f*(1 → 3)-α-D-Ara*f*-*O*-(CH_2_)_7_CH_3_ (acceptor A) and a branched trisaccharide [α-D-Ara*f*(1 → ]3,5-α-D-Ara*f*-*O*-(CH_2_)_7_CH_3_ (acceptor B). By implementing a well established AraT assay (Birch et al., 2008, Lee et al., 1997) we first assessed the array of products formed from incubating acceptor A and acceptor B (independently) with p[^14^C]Rpp and membranes prepared from *C. glutamicum* and each mutant strain, supplemented with decaprenol monophosphate. Assays were conducted both in the absence and presence of EMB in order to inhibit the single Emb protein which, under standard assay conditions, accounts for the majority of 5-Ara*f* residues deposited into endogenous AG precursors as well as neo-glycolipid acceptors (Birch et al., 2008). Following organic solvent extraction and separation of [^14^C]-labelled reaction products by TLC (from acceptor A), autoradiography revealed the presence of two bands that migrate to a Rf of 0.3 or 0.41, which we labelled product A^1^ and product A^2&3^, respectively (Fig. 3A). For acceptor B, a similar profile was observed with two bands either migrating to a Rf of 0.24 or 0.35, which we labelled product B^1^ and product B^2&3^, respectively (Fig. 4A). Assays conducted using wild type membranes and either acceptor A or B, produce a TLC autoradiogram with a product profile comprised of a single band annotated as product A^1^ and product B^1^, respectively, and is unaltered when EMB is included in the reaction mix (Figs. 3A and 4A). This combination of bands visible on the TLC changes significantly when assays are repeated using membranes devoid of AftB activity, suggesting that the slower migrating products (product A^1^ and product B^1^) produced from wild type *C. glutamicum* membranes contain [^14^C]Ara*f*-β(1 → 2) residues (Seidel et al., 2007a) by the AraT activity of AftB which dominates under standard assay conditions (Figs. 3AB and 4AB). The faster migrating bands, which appear in assays conducted with membranes prepared from either *C. glutamicum*Δ*aftB* or *C. glutamicum*Δ*aftB*Δ*aftD* (irrespective of whether acceptor A or acceptor B was used), have been labelled as product A^2&3^ (for acceptor A) and product B^2&3^ (for acceptor B). It is clear that for both of the acceptors (A and B) used in these assays, the formation of the resultant product migrating fastest on the TLC is sensitive to EMB suggesting that its appearance is, in part, due to the presence of the single Emb AraT and therefore contains an [^14^C]Ara*f*-α(1 → 5) residue (product A^2^ and B^2^) attached to both of the acceptors in question (Figs. 3AB and 4AB). However, assays conducted using membranes prepared from *C. glutamicum*Δ*aftB* supplemented with EMB produces a faintly visible band migrating to the same position on the TLC to assays conducted in the absence of EMB (hence the annotation of this band as product A^2&3^), suggesting the existence of an additional residual [^14^C]Ara*f*-α(1 → 5) AraT activity which is insensitive to EMB (product A^3^) (Fig. 3AB). Interestingly, assays conducted with membranes devoid only of AftD produce an identical TLC profile to that of the wild type assays, which is due to the “dominant” endogenous AraT activity of AftB, even in the presence of EMB (Figs. 3A and 4A). We have previously observed this dominant AftB activity when performing glycosyltransferase assays using similar neoglycolipid acceptors (Birch et al., 2008, Lee et al., 1997, Seidel et al., 2007a). In addition to the α(1 → 5) AraT activity of Emb, these radiolabelled assays provide indicative evidence for the presence of another α(1 → 5) AraT (*vis a vis* to product A^3^ and B^3^) endogenous to *C. glutamicum*.

### Evidence showing AftD exerts α(1 → 5) arabinofuranosyltransferase activity

In an effort to assign AftD with corresponding biochemical function, in terms of specific arabinofuranosyltransferase activity, we repeated scaled-up AraT assays using non-radiolabelled substrates. This experimental approach enabled production of sufficient quantities of reaction products to allow for subsequent chemical characterisation *via* GC/MS and electrospray mass spectrometry (ES-MS). Our attempts to extract individual bands (in isolation) from preparative TLCs were thwarted due to the technically challenging nature of closeness in retardation factors of the reaction products. Therefore, we analysed the “total pool of reaction products” in order to determine what array of glycosidic linkages resulted from the incubation of either acceptors A and B with the various strains of *C. glutamicum* membrane preparations. Assays conducted using acceptor A and *C. glutamicum* membranes resulted in the formation of at least three products made possible by the endogenous β(1 → 2) and α(1 → 5) AraT activities of AftB, Emb and AftD respectively (Fig. 3B), which upon addition of EMB, induced a complete loss in the formation of 3,5-Ara*f* (Fig. 3C). ES-MS analysis of per-*O*-methylated products resulted in a major peak at m/z of 647 (624 + Na) indicating that all products extracted exist as a trisaccharide (data not shown). As expected, when membranes from *C. glutamicum*Δ*aftB* were used in combination with acceptor A, all residual β(1 → 2) activity was abolished due to the removal of the AftB enzyme (Seidel et al., 2007a) (Fig. 3C). Chemical analysis of reaction products resulting from assays repeated in the presence of EMB revealed a linkage profile that fits a situation in which one can only conclude that a single α(1 → 5) Ara*f* has been incorporated into the acceptor substrate (Fig. 3B and C). Importantly, this product corresponds to an α-D-Ara*f*(1 → 5)-α-D-Ara*f*(1 → 3)-α-D-Ara*f*-*O*-(CH_2_)_7_CH_3_ trisaccharide product (product A^3^), which is also confirmed by a single ion at an m/z of 647 (624 + Na) (data not shown). Interestingly, the peak corresponding to 5-Ara*f* is missing from the *C. glutamicum*Δ*aftD* assay products (product A^3^) with an additional loss of 3,5-Ara*f* when assays are repeated in the presence of EMB (Fig. 3C). Under these experimental conditions, the absence of AftD activity due to the deletion of *aftD* in combination with the chemically induced inactivation of Emb by EMB, only results in the formation of product A^1^ catalysed by AftB (Fig. 3B and C). The AraT activity of Emb can be clearly observed in the results obtained from experiments carried out using membranes isolated from *C. glutamicum*Δ*aftB*Δ*aftD*, which corresponds to the formation of product A2. A repetition of these assays supplemented with EMB revealed a “blank” chromatogram with a linkage profile comprised only of background “noise” and can be attributed to a nullification of all AraT activity, either as a result of genetic deletion or chemical inhibition by EMB (Fig. 3C). This expected result is also in accordance with the radiolabelled experiments previously described (Fig. 3A).

Each of the assays described above were, in essence, repeated like-for-like using acceptor B. This 3,5-Ara*f* branched tri-arabinoside neo-glycolipid acceptor was used to determine what the effect an additional α(1 → 5) Ara*f* residue had on the resultant product profiles obtained from cell free assays conducted using membranes produced from each of the *C. glutamicum* strains under investigation. Assays conducted using acceptor B and *C. glutamicum* membranes resulted in the formation of at least three major products (Fig. 4B) arising from endogenous β(1 → 2) and α(1 → 5) AraT activities of AftB, Emb and AftD respectively, which upon addition of EMB, caused a reduction in the peak corresponding to 5-Ara*f* (Fig. 4C). ES-MS analysis of per-*O*-methylated products resulted in two dominant ions appearing at m/z 807 (784 + Na) and 967 (944 + Na) indicating that the products extracted consist of a pool of tetrasaccharides and pentasaccharides (data not shown). As expected, when membranes from *C. glutamicum*Δ*aftB* were used in combination with acceptor B, all endogenous β(1 → 2) activity was abolished due to the removal of the AftB enzyme (Seidel et al., 2007a) (Fig. 4C). Chemical analysis of reaction products resulting from assays repeated in the presence of EMB revealed a linkage profile that fits a situation in which one can only conclude that a single α(1 → 5) Ara*f* has been incorporated into the acceptor substrate (Fig. 4B and C). Whilst we cannot unequivocally claim that this α(1 → 5) Ara*f* residue has been added to the 3-arm branch of acceptor B, the evidence gathered so far suggest that this is the likely position of insertion which corresponds to a tetrasaccharide product (product B^3^), which is also confirmed by a single ion at an m/z 807 (784 + Na) (data not shown). Interestingly, the peak corresponding to 5-Ara*f* is reduced in the *C. glutamicum*Δ*aftD* assay products and when assays are repeated in the presence of EMB this peak is completely abolished (Fig. 4C). Under these experimental conditions, the absence of AftD activity due to deletion of *aftD* in combination with the chemically induced inactivation of Emb by the front line drug EMB, only results in the formation of product B^1^ catalysed by AftB (Fig. 4A and B). The AraT activity of Emb can be clearly observed in the results obtained from experiments carried out using membranes isolated from *C. glutamicum*Δ*aftB*Δ*aftD*. This product profile closely resembles that of assays carried out using *C. glutamicum*Δ*aftB* supplemented with EMB and therefore lends supporting evidence that Emb is responsible for the formation of product B^2^ (Fig. 4A and B). A repetition of these assays supplemented with EMB again reveals a “blank” chromatograph with a linkage profile comprised only of background “noise” (Fig. 4C).

**Fig. 5:**
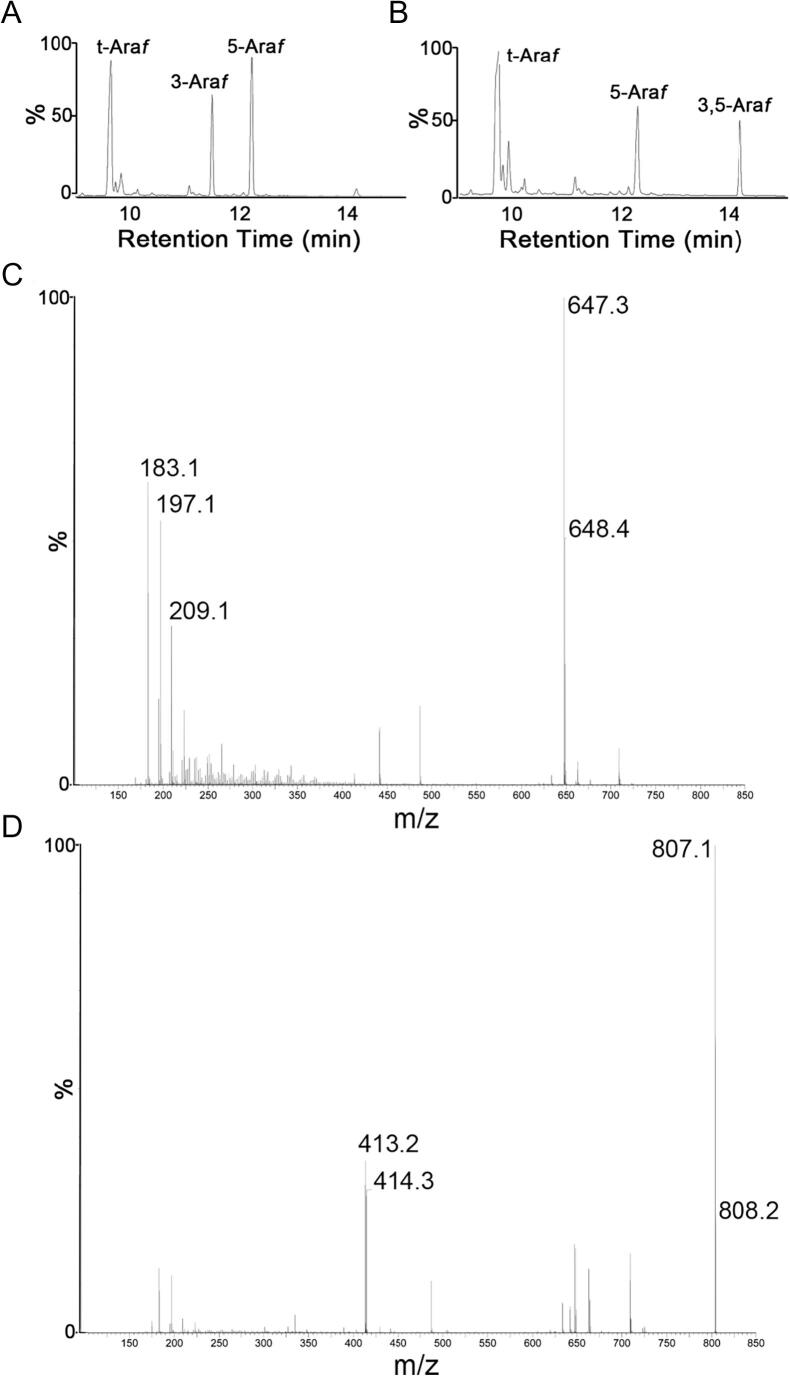
GC/MS and ES-MS characterisation of *in vitro* synthesized reaction products from the arabinofuranosyltransferase assays utilizing both acceptors A and B. *A*, GC/MS analysis and *B*, ES-MS analysis of products derived from assays using acceptor A and membranes prepared from *C. glutamicum*Δ*aftB*Δ*aftD* over-expressing Mt-*aftD* supplemented with EMB. *C*, GC/MS analysis and *D*, ES-MS analysis of products derived from assays using acceptor B and membranes prepared from *C. glutamicum*Δ*aftB*Δ*aftD* over-expressing Mt-*aftD* supplemented with EMB.

As part of this investigation we wanted to investigate the biochemical function of the *M. tuberculosis* ortholog of AftD. Our attempts to study Mt-AftD as recombinant protein have been hampered due to the extensive hydrophobic nature of this protein. Therefore, in order to study the biochemistry of Mt-AftD, we made further use of the previously described cell free assay which contains membranes prepared from *C. glutamicum*Δ*aftB*Δ*aftD* containing Mt-AftD *via* the aid of an over expression plasmid. In this instance, all isolated membranes are devoid of residual AftB and Cg-AftD activity and the α(1 → 5) Ara*f* transferase activity of Emb was abolished by supplementing all assay reactions with EMB. This created an experimental situation which enabled us to make a direct study of Mt-AftD expressed into a native *Corynebacteriales* membranous system. When assays were carried out using acceptor A, chemical analysis of the products by GC/MS revealed a linkage profile (Fig. 5A) almost identical to that of the products being formed from the previously observed *C. glutamicum*Δ*aftB* + EMB (Fig. 3BC). ES-MS analysis of this product revealed a major ion at 647 m/z (Fig. 5C), suggesting that this trisaccharide product is in fact α-D-Ara*f*(1 → 5)-α-D-Ara*f*(1 → 3)-α-D-Ara*f*-*O*-(CH_2_)_7_CH_3_ (product A^3^, Fig. 3B). When assays were carried out using acceptor B, chemical analysis of the products by GC/MS revealed a linkage profile (Fig. 5B) almost identical to that of the products being formed from the previously observed *C. glutamicum*Δ*aftB* + EMB (Fig. 4BC). ES-MS analysis of this product revealed a major ion at 807 m/z (Fig. 5D), suggesting that this tetrasaccharide product is in fact α-D-Ara*f*(1 → 5) extension of the 3-arm of acceptor B yielding a product B^3^ (Fig. 4B).

## Discussion

During our earlier studies, we discovered the role of AftA (Alderwick et al., 2006b), AftB (Seidel et al., 2007a) and AftC (Birch et al., 2008) which has shed new light on how members belonging to the *Corynebacteriales* (such as *C. glutamicum* and *M. tuberculosis*) synthesise their cell walls. We conducted a bioinformatic analysis of a variety of *Corynebacteriales* genomes looking specifically for ORFs encoding for GT-C glycosyltransferases. Many of these ORFs have now been assigned a function, either belonging to AG biosynthesis and/or LM/LAM (Abrahams and Besra, 2016, Jankute et al., 2015). Apart from Rv0051, which remains to be assigned its proper function, Rv0236c (AftD) presented itself as a potential candidate to be involved in the assembly of the *M. tuberculosis* cell wall. We speculated that AftD might encode for a GT-C glycosyltransferase that provided α(1 → 5) arabinofuranosyltransferase activity, possibly elongating the 3-arm of the arabinan domain in a processive manner. Contrary to the findings reported by Skovierova et al. (2009) our chemical analysis of the *C. glutamicum*Δ*aftD* cell wall revealed a clear phenotype. The main difference being a ∼1.5-fold increase in the amount of TMCM deposited into the cell wall with a corresponding 7% decrease in arabinogalactan-related cell wall arabinose. The severity of this change in cell wall phenotype is exacerbated when both *aftB* and *aftD* are deleted in *C. glutamicum* and further glycosidic linkage analysis by GC/MS and HSQC 2D-NMR provides evidence that AftD is involved in formation of AG in *C. glutamicum*. Specifically, whilst deletion of *aftD* causes a slight reduction in the amount of 5-Ara*f* relative, a double deletion of *aftB* and *aftD* induces additional and unique alterations to the glycosidic linkage profiles manifested by the appearance of a 3-Ara*f* residue. This motif can only appear in a situation whereby a terminal Ara*f* residue is positioned on the 3-OH of a preceding Ara*f* residue on the arabinan polymer. Skovierova et al. (2009) demonstrated that AftD from *M. smegmatis* displays α(1 → 3)-Ara*f* transferase activity. Whilst we fully acknowledge the findings of these authors, this study now provides new evidence demonstrating that both Cg-AftD and Mt-AftD exhibit α(1 → 5)-Ara*f* transferase activity.

**Fig. 6:**
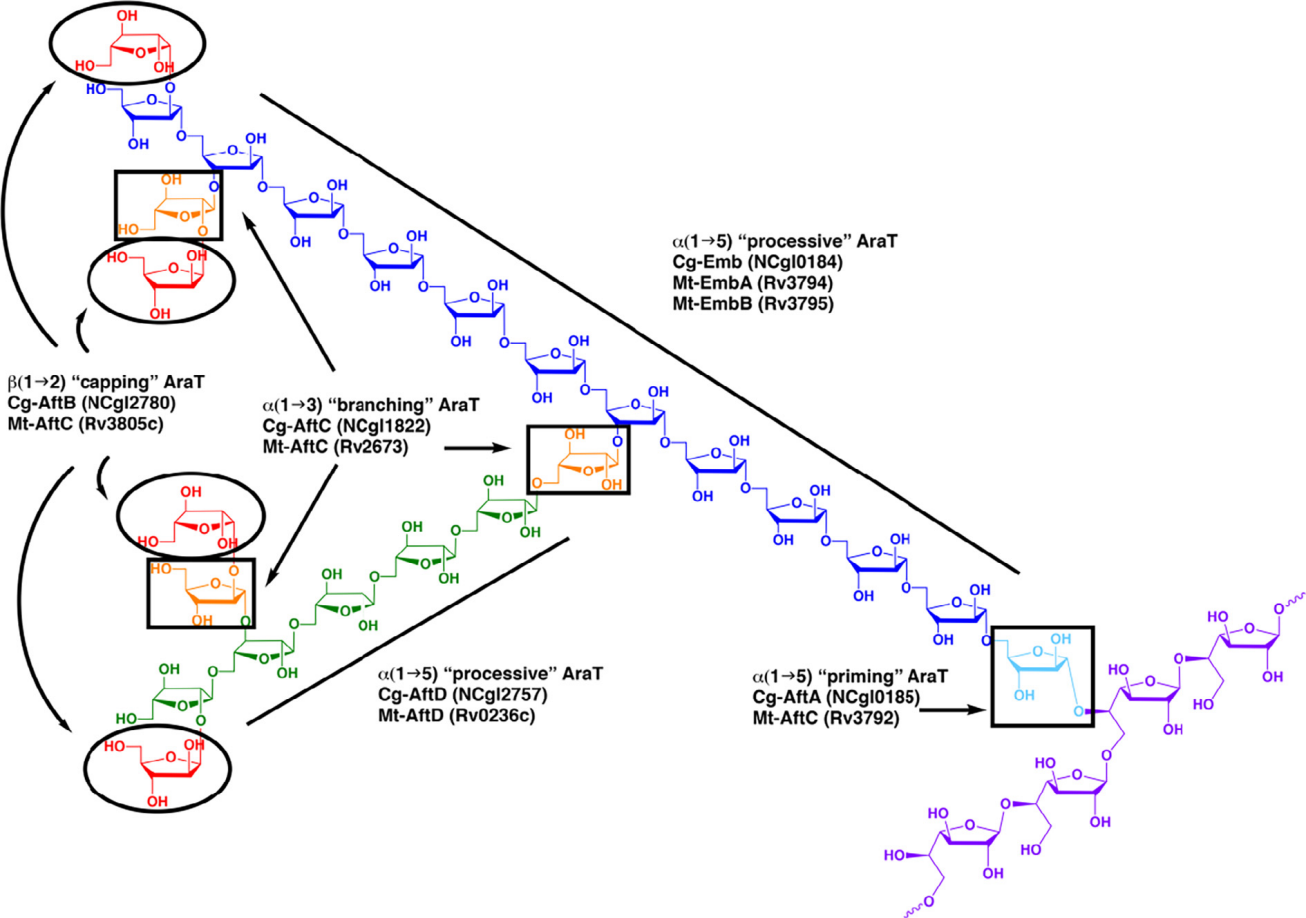
Revised pathway of arabinan biosynthesis illustrating the individual roles of Emb, AftA, AftB, AftC and AftD arabinofuranosyltransferases.

By taking a review of all publications (post-2005) concerning the biosynthesis of the mycobacterial cell wall, including the findings of this report, only now are we able to fully appreciate the intricate mechanisms by which this pathogen assembles its cell wall (Fig. 6). The galactan domain of AG is first synthesised within the cytoplasm of the cell, which is in turn conjugated to decaprenol pyrophosphate *via* a unique GlcNAc-Rha linker unit. Once this linear polysaccharide has been translocated across the cytoplasmic membrane (Dianiskova et al., 2011), AftA inserts three Ara*f* residues at the 8th, 10th and 12th position of the galactan domain (Alderwick et al., 2006b). In the case of *C. glutamicum*, the single Emb protein then serves to processively extend each of the “primed” Ara*f* residues with α(1 → 5)-Ara*f* residues to an optimal length (approximately 5–6 residues) before AftC adds an α(1 → 3)-Ara*f* unit. This sequence of events is most likely to be the case in *Mycobacterium spp.* but with either EmbA or EmbB providing α(1 → 5) AraT activity. The generation of a newly synthesised branched arabinan “motif” is a critical event leading to the bifurcation of the arabinan domain. Whilst the molecular mechanism of this requires further investigation, it is tempting to suggest that a branching of the arabinan oligosaccharide induces differential affinities between the acceptor substrate (K_m_) and the next AraT. In this regard, data from this study suggest that AftD recognises the 5-OH of a 3-Ara*f* and Emb (or EmbA or EmbB in the case of *Mycobacterial spp.*) recognises the 5-OH of the 5-Ara*f* of the main arabinan chain (Fig. 6). This mechanism has been demonstrated in the globular C-terminal domain of EmbC, which shows differential affinities for a variety of arabinan oligosaccharides that differ in length (Alderwick et al., 2011). By an as yet unknown mechanism, the AraTs are then able to distinguish between a variety of arabinan chain lengths by introducing a 3,5-Ara*f* residue before AftB completes arabinan biosynthesis by “capping” the 3,5-Ara*f* motif with two terminal β(1 → 2) residues (Fig. 6). The structural complexity of mycobacterial D-arabinan is mirrored in the equally complex biosynthetic systems that have evolved to assemble this crucial cell wall molecule. We have shown that many of these glycosyltransferases interact with each other in a combination of homo and hetero oligomeric complexes (Jankute et al., 2014). For instance, both AftA and AftB directly interact with Emb and AftC, likely forming a large membrane bound multi-enzyme complex (Jankute et al., 2014). Interestingly, our analysis of AG prepared from the *C. glutamicum*Δ*aftB*Δ*aftD* mutant in this study reveals a significant increase in 5-Ara*f* glycosyl linkages. In this situation, due to complete absence of β(1 → 2)-Ara*f* and α(1 → 5)-Ara*f* activity (from AftB and AftD, respectively), extension of the D-arabinan is reliant upon the remaining endogenous α(1 → 3)-Ara*f* and α(1 → 5)-Ara*f* transferase activities from AftC and Emb, respectively. We speculate that this phenotype could be partly rationalised by disruption multi-enzyme complex that work in concert to synthesise mycobacterial D-arabinan. Further studies are needed to investigate the precise molecular mechanisms by which all the variants of the AraT family of enzymes work in concert to produce AG, which is crucial to the structural integrity of the mycobacterial cell wall.

## Significance

One of the greatest burdens on humanity is the continued rise and prevalence of TB. New chemotherapeutic agents are urgently required to combat this human pathogen especially with the advent of multi-drug-resistant (MDR) and extensively-drug-resistant (XDR) strains of *M. tuberculosis*. Arabinogalactan (AG) is an essential cell wall molecule and its formation is targeted by treating TB patients with the front line drug, ethambutol. This study focuses on AftD, an essential enzyme in this pathway. Through a comprehensive phenotypic characterisation of both single and double mutants of *C. glutamicum* devoid of *aftD* and *aftB*, combined with detailed biochemical AraT assays, we have deconvoluted the molecular genetics of *aftD* in terms of its involvement in D-arabinan biosynthesis as an α(1 → 5)-Ara*f* transferase.
