# Supplementary material for: AftD functions as an α1 → 5 arabinofuranosyltransferase involved in the biosynthesis of the mycobacterial cell wall core
Source: Cell Surf. 2017 Dec 1;1:2–14. doi: 10.1016/j.tcsw.2017.10.001 (PMC6034362; doi:10.1016/j.tcsw.2017.10.001)
Supplement: Supplementary data 1 [file mmc1.docx]

**SUPPLEMENTAL INFORMATION**

AftD functions as a short-chain α1→5 processive arabinofuranosyltransferase involved in the biosynthesis of the mycobacterial cell wall core.

Luke J. Alderwick^1,*^, Helen L. Birch^1^, Karin Krumbach^2^, Michael Bott^2^, Lothar Eggeling^2^, and Gurdyal S. Besra^1,*^

^1^School of Biosciences, University of Birmingham, Edgbaston, Birmingham, B15 2TT, UK

^2^Institute for Biotechnology 1, Forschungszentrum Juelich, D-52425 Juelich, Germany

^*^Correspondence: [g.besra@bham.ac.uk](mailto:g.besra@bham.ac.uk) (G.S.B), [l.alderwick@bham.ac.uk](mailto:l.alderwick@bham.ac.uk) (L.J.A)

**SUPPLEMENTAL DATA**

**
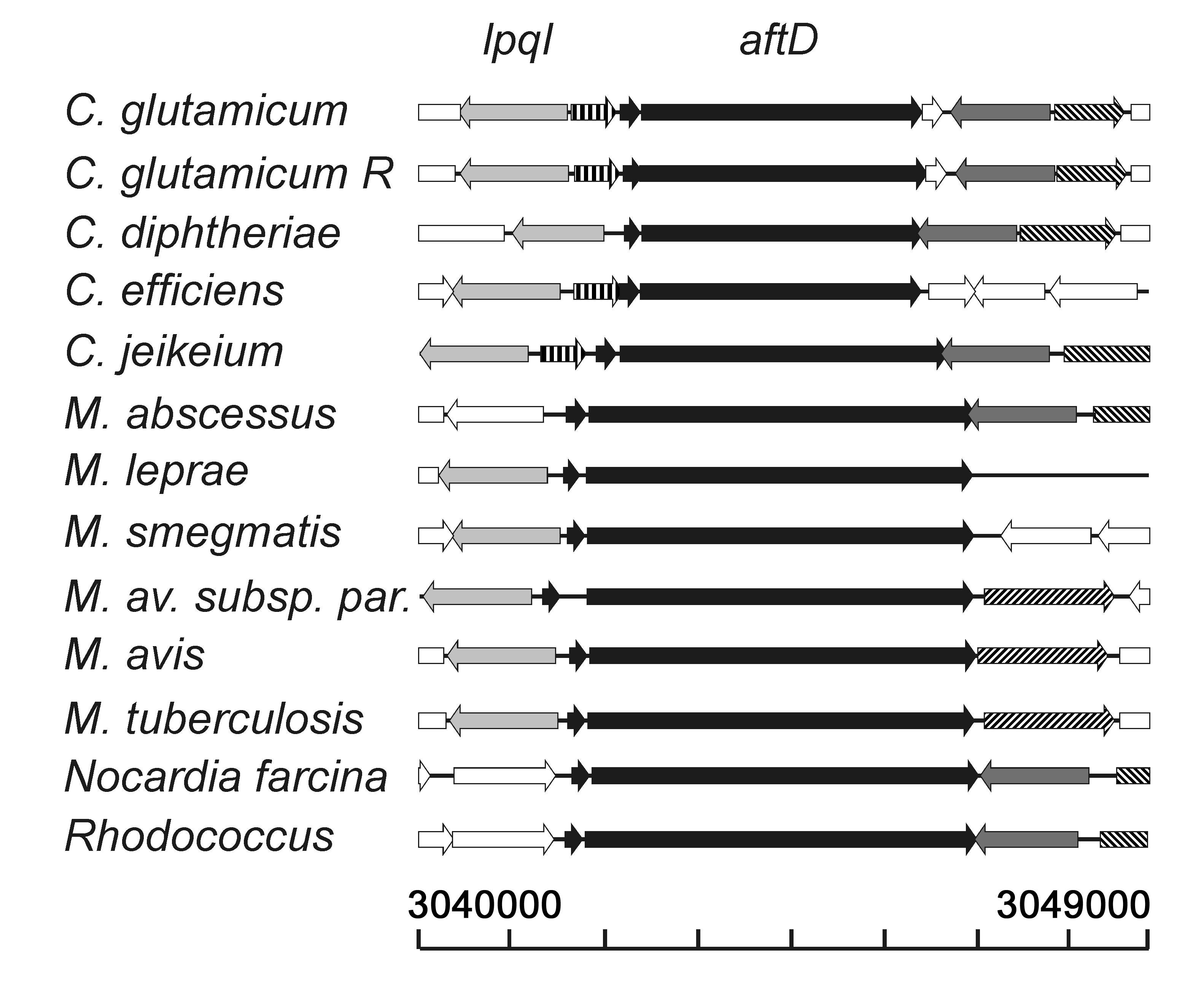
**

**Figure S1. Comparison of the *aftD* locus within the *Corynebacteriales.***

The locus in *C. glutamicum* consists of *aftD* (*NCgl2757*) with the two small upstream genes *NCgl2756* and *NCgl2755* probably forming an operon, as well as *lpqI* (NCgl2754). *AftD*, NCgl2756 and *lpqI* are retained in almost all *Corynebacteriales* with *lpqI* predicted to encode a secreted protein possessing the *O*-glycosyl hydrolase domain PF00933. Shown is an 8 kb region of the syntenic genomes with orthologous genes shaded accordingly, gene coordinates are given for the *C. glutamicum* ATTC 13032 genome. The abbreviations and strains used are as follows: *M., Mycobacterium*; *C., Corynebacterium;* and *M. av. p.*, *M. avium paratuberculosis*. *C. glutamicum*, *C. glutamicum* ATCC13032; *C. diphtheriae*, *C. diphtheriaeNCTC*-13129; C. *efficiens*, *C. efficiens* YS-314; *M. leprae*, *M. leprae* TN; *M. smegmatis*, *M. smegmatis* MC2; *M. tuberculosis*, *M. tuberculosis* H37Rv; *Nocardina farcina*, *Nocardina farcina* IFM10152; *Rhodococcus*, *Rhodococcus* sp. RHA1.

**Figure S2. The consequences of Cg-*aftD* deletion mutant (complemented with Cg-*aftD and* Mt-*aftD)* (A) and Cg-*aftB*/Cg-*aftD* double deletion mutant (complemented with Cg-*aftD and* Mt-*aftD)* (B) on growth in rich medium (BHI).** *A,* Growth of *C. glutamicum* (●), *C. glutamicum*Δ*aftD* (◼), *C. glutamicum*Δ*aftD* pVWEx-*Cg*-*aftD* (▲) and *C. glutamicum*Δ*aftD* pVWEx-*Mt*-*aftD* (△). *B,* Growth of *C. glutamicum* (●), *C. glutamicum*Δ*aftB*Δ*aftD* (◼),*C. glutamicum*Δ*aftB*Δ*aftD* pVWEx-*Cg*-*aftD* (▲) and *C. glutamicum*Δ*aftB*Δ*aftD* pVWEx-*Mt*-*aftD* (△).

**
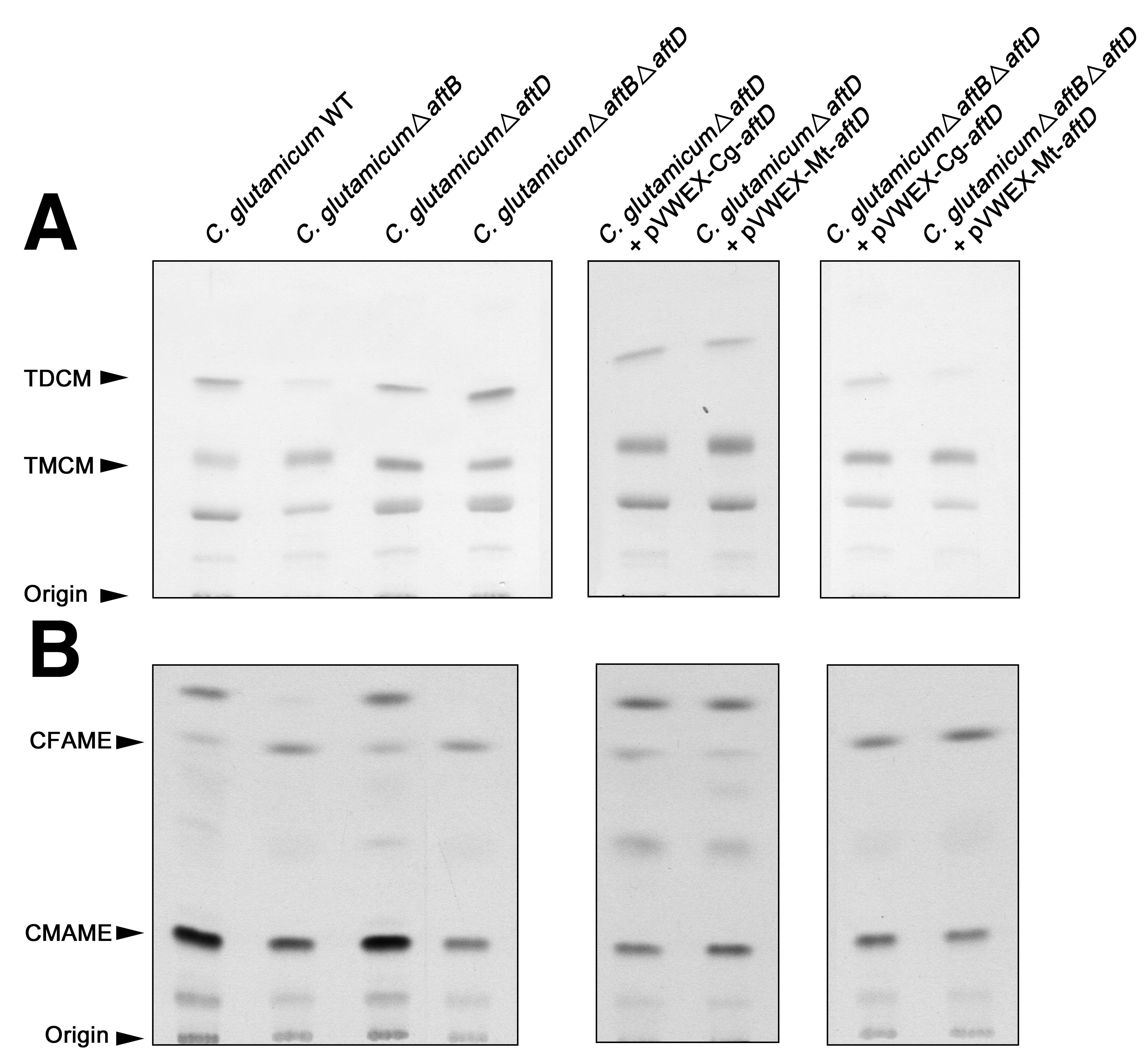
**

**Figure S3. Analysis of cell wall associated (A) and cell wall bound (B) coronomycolic acids from *C. glutamicum*, *C. glutamicum*Δ*aftB, C. glutamicum*Δ*aftD*, *C. glutamicum*Δ*aftB*Δ*aftD, C. glutamicum*Δ*aftD* pVWEX-Cg-*aftD* and *C. glutamicum*Δ*aftD* pVWEX-Mt-*aftD C. glutamicum*Δ*aftB*Δ*aftD* pVWEX-Cg-*aftD* and *C. glutamicum*Δ*aftB*Δ*aftD* pVWEX-Mt-*aftD*.** *A,* Extractable lipids were extracted using CHCl_3_:CH_3_OH:H_2_O (10/10/3; v/v/v/v) and subjected to TLC using silica gel plates (5735 silica gel 60F_254_, Merck) developed in CHCl_3_:CH_3_OH:NH_4_OH (80:20:2, v/v/v) to separate [^14^C]-labelled TDCM and TMCM. *B,* Cell wall bound lipids were released from de-lipidated cells by the addition of tetra-butylammonium hydroxide at 100°C overnight, and methylated. An equivalent aliquot from each strain was subjected to TLC using silica gel plates (5735 silica gel 60F_254_, Merck), and developed in petroleum ether/acetone (95:5, v/v) to separate [^14^C]-labelled to reveal CMAMEs and CFAMEs.

**
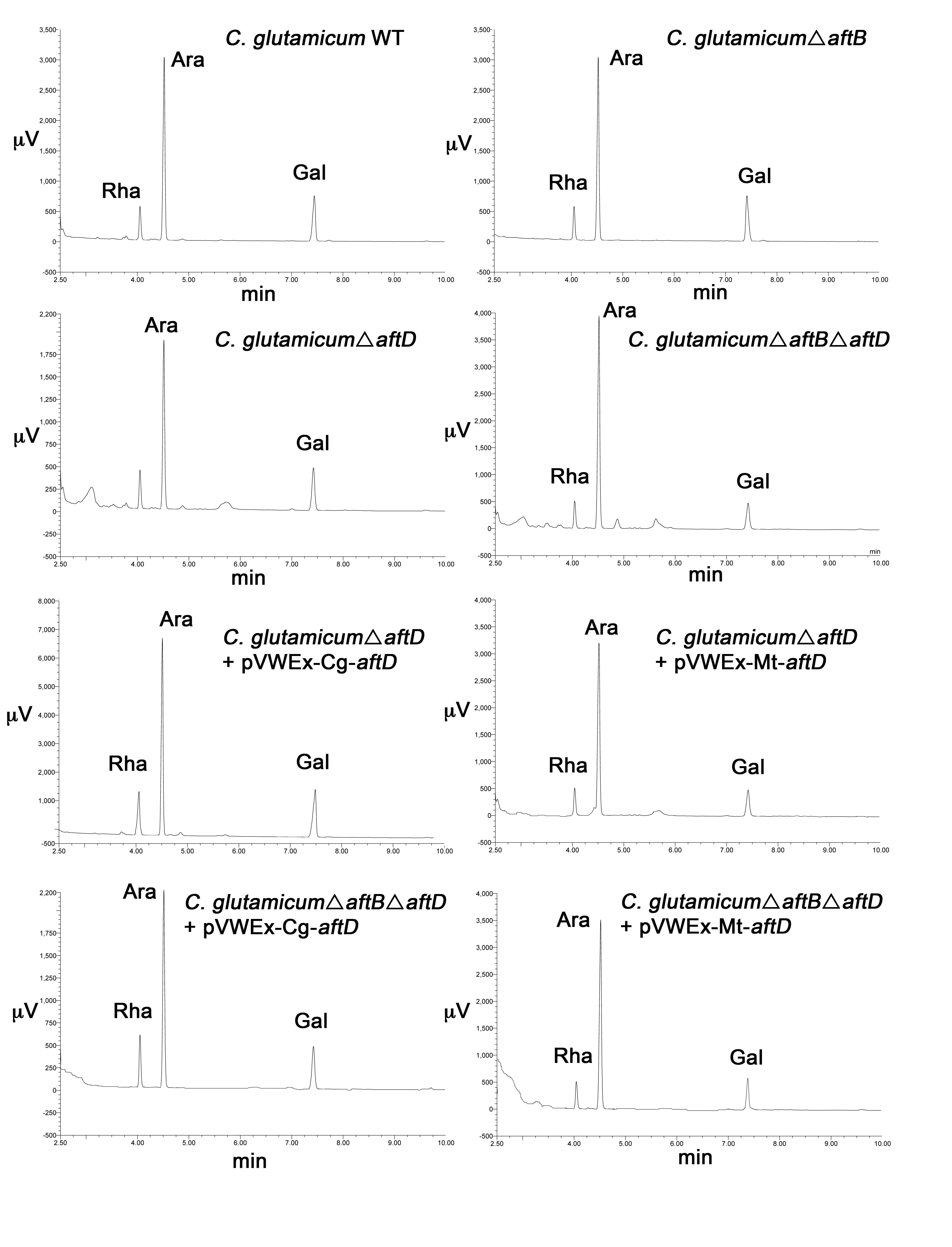
**

**Figure S4. Total sugar compositional analysis of *C. glutamicum*, *C. glutamicum*Δ*aftB, C. glutamicum*Δ*aftD*, *C. glutamicum*Δ*aftB*Δ*aftD, C. glutamicum*Δ*aftD* pVWEX-Cg-*aftD* and *C. glutamicum*Δ*aftD* pVWEX-Mt-*aftD C. glutamicum*Δ*aftB*Δ*aftD* pVWEX-Cg-*aftD* and *C. glutamicum*Δ*aftB*Δ*aftD* pVWEX-Mt-*aftD.*** Abbreviations: Ara, arabinose; Gal, galactose; Rha, rhamnose.


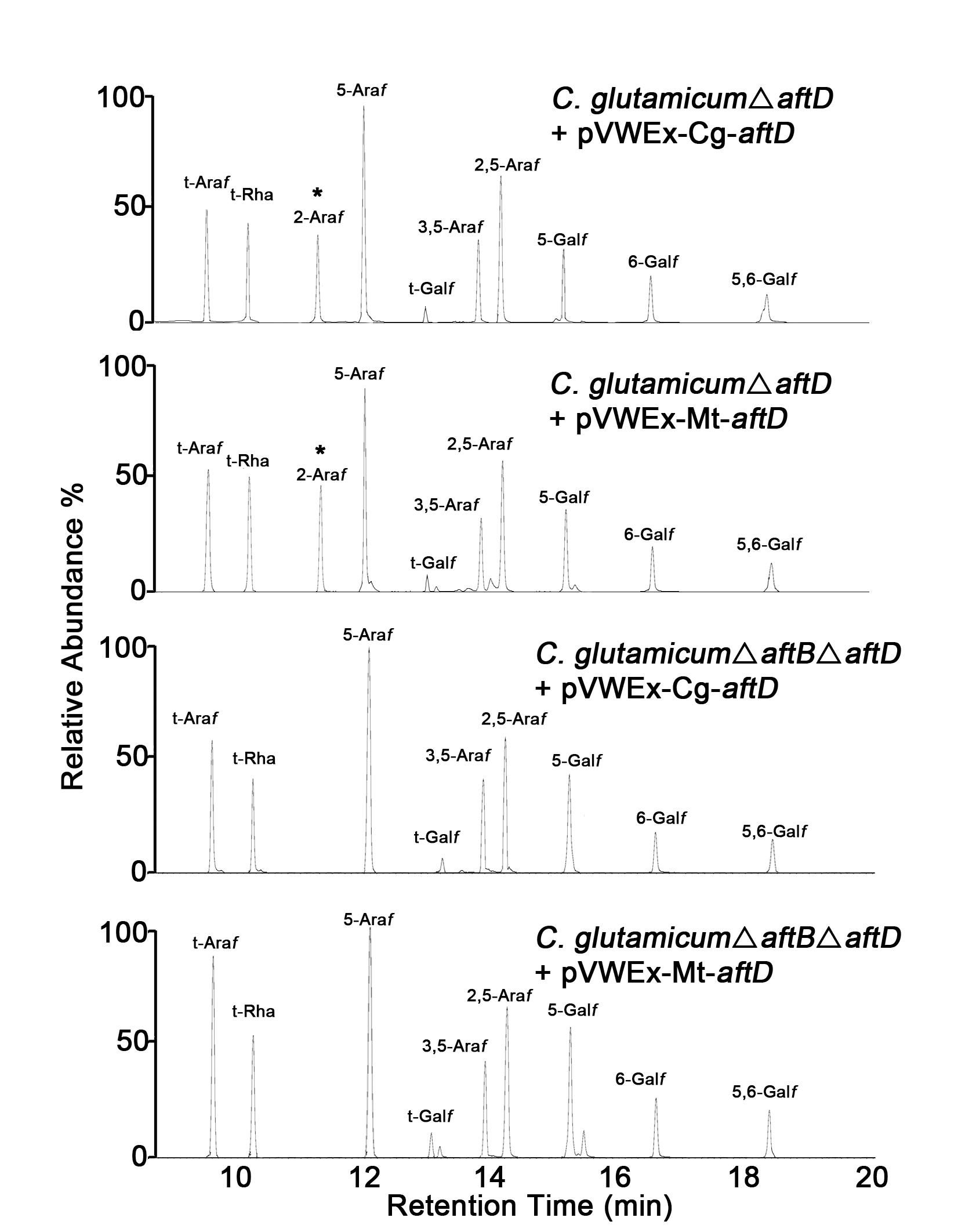


**Figure S5. Gas Chromatography/Mass Spectrometry (GC/MS) analysis of partially per-*O*-methylated, per-*O*-acetylated alditol acetate derivatives of purified arabinogalactan from *C. glutamicum*Δ*aftD* pVWEX-Cg-*aftD* and *C. glutamicum*Δ*aftD* pVWEX-Mt-*aftD C. glutamicum*Δ*aftB*Δ*aftD* pVWEX-Cg-*aftD* and *C. glutamicum*Δ*aftB*Δ*aftD* pVWEX-Mt-*aftD*.**.

**
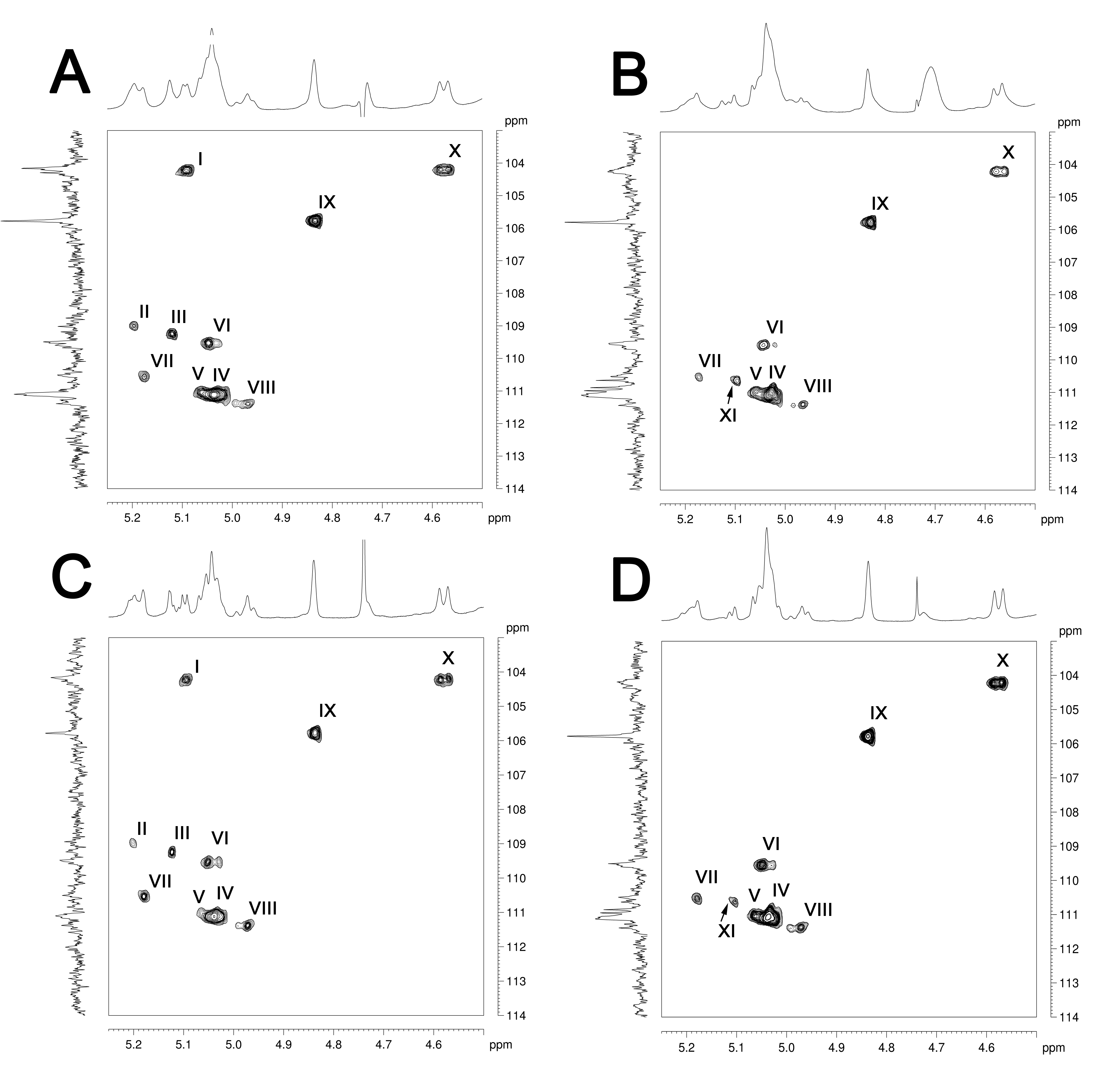
**

**Figure S6. Two-dimensional NMR spectra of arabinogalactan purified from *C. glutamicum* (A), *C. glutamicum*Δ*aftB* (B)*, C. glutamicum*Δ*aftD* (C) and *C. glutamicum*Δ*aftB*Δ*aftD* (D).** ^1^H, ^13^C HSQC NMR spectra were acquired in D_2_O at 313K. Expanded regions (δ ^1^H: 5.0-5.30, δ ^13^C: 101-111) are shown. *t*-β-Ara*f* (I), 2-α-Ara*f*→3 (II), 2-α-Ara*f*→5 (III), 5-α-Ara*f* (IV), 3,5-α-Ara*f* (V), 2,5-α-Ara*f* (VI), 5-β-Gal*f* (VII), 6-β-Gal*f* (VIII), *t*-α-Rha*p* (IX and X) and 3-α-Ara*f* (XI) are highlighted.

**SUPPLEMENTAL EXPERIMENTAL PROCEDURES**

**Extraction and analysis of cell wall coronomycolic acids.**

*C. glutamicum* cultures (5 ml) were grown and metabolically labelled at mid-logarithmic phase of growth using 1 μCi/ml [1,2-^14^C]acetate (50-62 mCi/mmol, GE Healthcare, Amersham Bioscience) for 4 h at 30°C with gentle shaking, harvested, washed and freeze-dried. Cells were then extracted by two consecutive extractions with 2 ml of CHCl_3_/CH_3_OH/H_2_O (10:10:3, v/v/v) for 4 h at 50°C to provide cell wall associated lipids and analyzed as described previously (Alderwick et al., 2006). The crude lipid extracts were resuspended in CHCl_3_:CH_3_OH (2:1) and equivalent aliquots (50,000 cpm) analyzed by TLC using silica gel plates (5735 silica gel 60F_254_, Merck) developed in CHCl_3_:CH_3_OH:NH_4_OH (80:20:2, v/v/v) to separate [^14^C]-labelled TDCM and TMCM (Alderwick et al., 2006). Lipids were visualized by autoradiography by overnight exposure of Kodak X-Omat AR film to the TLC plates to reveal labelled lipids, quantified by phosphorimaging and compared to known standards . The bound CMAMEs from the above de-lipidated extracts were released by the addition of 2 ml of 5% aqueous solution of tetra-butyl ammonium hydroxide followed by overnight incubation at 100°C. After cooling, water (2 ml), CH_2_Cl_2_ (4 ml) and CH_3_I (500 µl) were added and mixed thoroughly for 30 min. The lower organic phase was recovered following centrifugation and washed three times with water (4 ml), dried and resuspended in diethyl ether (4 ml). After centrifugation the clear supernatant was again dried and resuspended in CH_2_Cl_2_ (100 μl). An aliquot (5 μl) from each strain was subjected to scintillation counting and an equivalent (5 μl) aliquot analyzed by TLC using silica gel plates (5735 silica gel 60F_254_, Merck), developed in petroleum ether/acetone (95:5, v/v) and visualized by autoradiography by exposure of Kodak X-Omat AR film to the TLC plates to reveal [^14^C]-labeled CMAMEs compared to known standards (Gande et al., 2004).

**NMR spectroscopic analysis of arabinogalactan**

NMR spectra of LAM samples were recorded on a Bruker DMX-500 equipped, with a double resonance (1H/X)-BBi z-gradient probe head. All samples were exchanged in D_2_O (D, 99.97% from Euriso-top, Saint-Aubin, France), with intermediate lyophilization, and then dissolved in 0.5 ml D_2_O and analyzed at 313K. The ^1^H and ^13^C NMR chemical shifts were referenced relative to internal acetone at 2.225 and 34.00 ppm, respectively. All the details concerning NMR sequences used and experimental procedures were described previously (Birch et al., 2010).

**SUPPLEMENTAL REFERENCES**

Alderwick, L.J., Seidel, M., Sahm, H., Besra, G.S., and Eggeling, L. (2006). Identification of a Novel Arabinofuranosyltransferase (AftA) Involved in Cell Wall Arabinan Biosynthesis in *Mycobacterium tuberculosis*. The Journal of biological chemistry *281*, 15653-15661.

Birch, H.L., Alderwick, L.J., Appelmelk, B.J., Maaskant, J., Bhatt, A., Singh, A., Nigou, J., Eggeling, L., Geurtsen, J., and Besra, G.S. (2010). A truncated lipoglycan from mycobacteria with altered immunological properties. Proceedings of the National Academy of Sciences of the United States of America *107*, 2634-2639.

Gande, R., Gibson, K.J., Brown, A.K., Krumbach, K., Dover, L.G., Sahm, H., Shioyama, S., Oikawa, T., Besra, G.S., and Eggeling, L. (2004). Acyl-CoA carboxylases (accD2 and accD3), together with a unique polyketide synthase (Cg-pks), are key to mycolic acid biosynthesis in *Corynebacterianeae* such as *Corynebacterium glutamicum* and *Mycobacterium tuberculosis*. The Journal of biological chemistry *279*, 44847-44857.
